# Targeted genome editing of plants and plant cells for biomanufacturing

**DOI:** 10.1007/s11248-021-00236-z

**Published:** 2021-03-01

**Authors:** J. F. Buyel, E. Stöger, L. Bortesi

**Affiliations:** 1 Fraunhofer Institute for Molecular Biology and Applied Ecology IME, Forckenbeckstrasse 6, 52074 Aachen, Germany; 2 Institute for Molecular Biotechnology, RWTH Aachen University, Worringerweg 1, 52074 Aachen, Germany; 3 Department of Applied Genetics and Cell Biology, University of Natural Resources and Life Sciences, Vienna, Austria; 4 Aachen-Maastricht Institute for Biobased Materials (AMIBM), Maastricht University, Brightlands Chemelot Campus, Urmonderbaan 22, 6167 RD Geleen, The Netherlands

**Keywords:** Metabolic optimization, Chassis, Modified glycosylation, Plant molecular farming, Programmable growth and senescence, Protease inactivation

## Abstract

Plants have provided humans with useful products since antiquity, but in the last 30 years they have also been developed as production platforms for small molecules and recombinant proteins. This initially niche area has blossomed with the growth of the global bioeconomy, and now includes chemical building blocks, polymers and renewable energy. All these applications can be described as “plant molecular farming” (PMF). Despite its potential to increase the sustainability of biologics manufacturing, PMF has yet to be embraced broadly by industry. This reflects a combination of regulatory uncertainty, limited information on process cost structures, and the absence of trained staff and suitable manufacturing capacity. However, the limited adaptation of plants and plant cells to the requirements of industry-scale manufacturing is an equally important hurdle. For example, the targeted genetic manipulation of yeast has been common practice since the 1980s, whereas reliable site-directed mutagenesis in most plants has only become available with the advent of CRISPR/Cas9 and similar genome editing technologies since around 2010. Here we summarize the applications of new genetic engineering technologies to improve plants as biomanufacturing platforms. We start by identifying current bottlenecks in manufacturing, then illustrate the progress that has already been made and discuss the potential for improvement at the molecular, cellular and organism levels. We discuss the effects of metabolic optimization, adaptation of the endomembrane system, modified glycosylation profiles, programmable growth and senescence, protease inactivation, and the expression of enzymes that promote biodegradation. We outline strategies to achieve these modifications by targeted gene modification, considering case-by-case examples of individual improvements and the combined modifications needed to generate a new general-purpose “chassis” for PMF.

## Significance statement

The review examines a number of diverse technologies for the production of recombinant proteins and small molecules in plant-based systems. The article emphasizes strain development, biomass accumulation and processing at the molecular, cellular and organism levels, thus providing the reader not only with an overview of the latest developments in plant genome editing, but also with decision-making tools for specific applications. The article also illustrates how genome editing and conventional transgenesis technologies can complement each other to establish platform host plants for biomanufacturing applications.

**P. Christou,** *University of Lleida-Agrotencio Center, Lleida and ICREA, Barcelona, Spain*.

## Introduction

Plants have provided humans with food, fiber, construction materials and useful chemicals for thousands of years. However, since the early 1990s they have also been promoted as production platforms (Hiatt et al. 1989), mostly to manufacture small molecules and recombinant proteins (Fischer and Buyel 2020). This niche area has expanded with the global bioeconomy starting around 2010 to include chemical building blocks, polymers and renewable energy (Buyel 2018). All these applications can be considered under the umbrella of “plant molecular farming” (PMF).

**Fig. 1:**
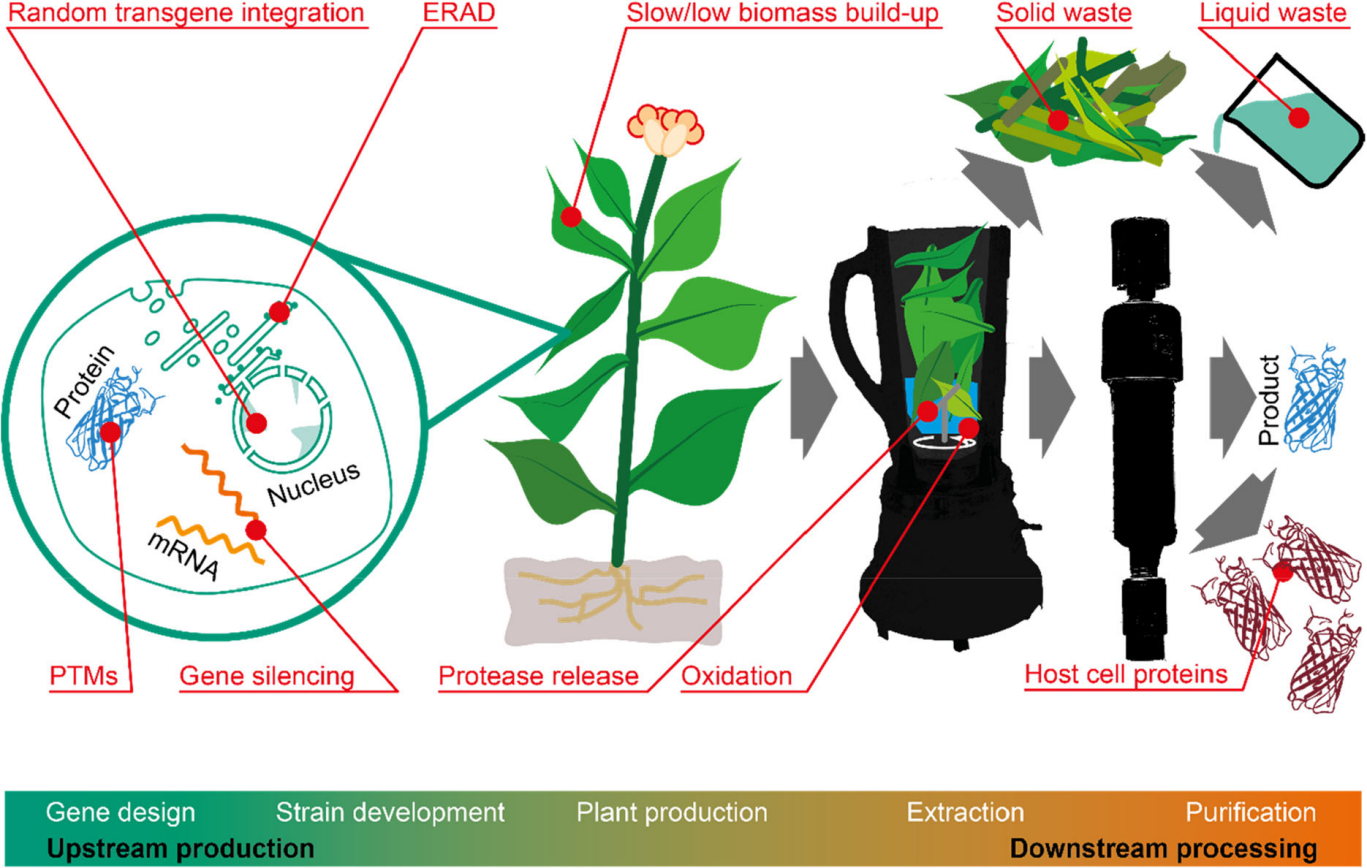
Potential limitations of plant-based processes for the industry-scale manufacturing of recombinant proteins. Several features of plants that affect their use as bioreactors and reduce the efficiency of the production process could be modified by genome editing to improve product quality, overall productivity and cost-efficiency. *ERAD* endoplasmic reticulum-associated degradation, *PTMs* post-translational modifications

Despite its potential to tap into alternative, renewable resources for manufacturing, PMF has yet to be adopted by the biomanufacturing industry on a large scale. Initially this reflected the uncertain regulatory framework, particularly for pharmaceuticals produced in intact plants (Fischer et al. 2012), as well as the limited understanding of associated process costs, and the lack of trained personnel and suitable manufacturing capacity (Alam et al. 2018; Walwyn et al. 2015; Buyel and Fischer 2012). However, an additional important drawback is the limited adaptation of plants and plant cells to the requirements of industry-scale manufacturing (Fig. 1). For example, targeted genetic manipulation in yeast has been common practice since the 1980s (Green and Tibbetts 1980), whereas efficient site-directed mutagenesis in higher plants only become available following the advent of CRISPR/Cas9 and similar genome editing technologies (Doudna and Charpentier 2014; Li et al. 2014). Industry has also been discouraged by the timeframe of 6–18 months needed to regenerate stable, transgenic plants (Sack et al. 2015) and the bottlenecks along the path to regulatory approval (Ma et al. 2015; Tusé et al. 2020). Furthermore, the highest level of product accumulation recorded in plants and plant cells is currently ~ 4 g kg^−1^ for GFP and similar levels have been achieved for monoclonal antibodies and influenza antigens as well (Yamamoto et al. 2018; Shoji et al. 2012; Zischewski et al. 2015) whereas mammalian cells often achieve yields > 25 g L^−1^ with well-characterized products such as antibodies (Yang et al. 2016). Product yields in plants can also vary substantially within the biomass (Sack et al. 2015; Buyel and Fischer 2012; Knödler et al. 2019), especially if host reactions, such as the response to infiltrating bacteria during transient expression, lead to the activation of endogenous proteases (Grosse-Holz et al. 2017). Proteins expressed in plants and plant cells gain non-human glycosylation profiles (Fischer et al. 2018; Strasser 2016), and further unwanted product modifications or degradation may occur during downstream processing (DSP) due to oxidation or proteolysis in the crude extract. DSP in general can be difficult to develop and operate for plant-based systems due to the large quantities of host cell proteins (HCPs) and potentially toxic metabolites in the extracts, including nicotine if whole tobacco plants are used as the production host (Buyel et al. 2015b). Finally, some plants and plant cells feature unfavorable characteristics for bioprocessing, such as a large but biosynthetically inactive vacuole that reduces volumetric and fresh mass-based productivity, and biomass components such as stems that accumulate very little product but are typically processed along with leaves to simplify the harvesting method. These factors add to the cost of goods during DSP and generate residual biomass that must be processed before disposal.

**Fig. 2:**
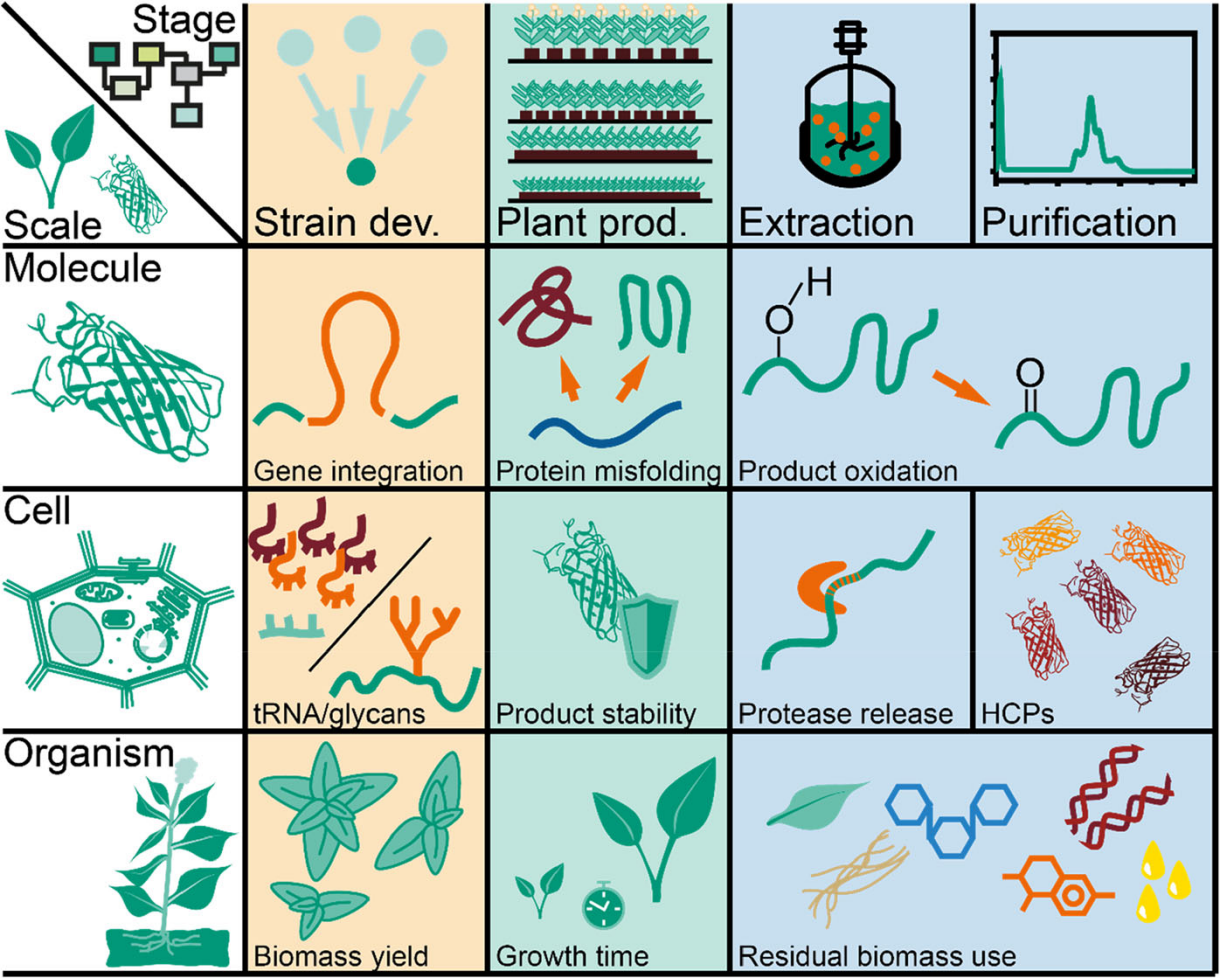
Applications of genetic engineering and genome editing to improve plant molecular farming depending on the process stages (columns) and scales (rows). Every step during process development (columns) can benefit at the molecular, cellular and organism levels (rows). The aim of the improvements is to increase product yields, achieve authentic or compatible post-translational modifications, and integrate the use of residual biomass

In this article, we discuss modifications that can help to improve plants for biomanufacturing applications, focusing on the production of recombinant proteins (Fig. 2). These modifications can be achieved by genetic engineering and/or genome editing, which provide complementary toolsets. We use the bottlenecks described above to illustrate progress that has already been made, and discuss potential improvements at the molecular, cellular and organism levels. First, we review the benefits of targeted gene integration platforms to design engineered plants before discussing modifications at the cellular level that can help to create a supportive environment for recombinant protein synthesis, including adaptations of the endomembrane system and modified glycosylation patterns. We then assess the options to modify HCP and metabolite profiles for streamlined DSP. Finally, we consider alterations on the whole-plant level such as growth habit and residual biomass processing. We conclude with our vision of how these improvements can be combined into a new general purpose “chassis” for PMF.

## Targeted gene integration platforms

### Landing pads for the rapid production of transgenic lines expressing multigene pathways

Conventional plant transformation (usually mediated by *Agrobacterium tumefaciens* or particle bombardment) generates random transgene insertion events. The inability to control the integration process leads to variable expression levels due to position effects and different transgene copy numbers. Position effects include transgene integration in genomic regions with different chromatin structures (active euchromatin vs. transcriptionally inactive heterochromatin) and in the proximity of native regulatory elements. Furthermore, epigenetic modifications such as methylation at the integration site can reduce the long-term stability of transgene expression, causing the productivity of selected lines to fall over time (Rajeevkumar et al. 2015). For these reasons, many independent transgenic events must be generated and screened to identify those with the highest expression levels, which is both time consuming and expensive.

The challenges of random transgene integration can be overcome using site-specific nucleases (SSNs). By introducing a double-strand break (DSB) at a pre-determined sequence, SSNs enable controlled transgene integration. Compared to SSN-induced indel formation, site-specific DNA insertion remains challenging because it is not the preferred outcome of DSB repairs in plants. SSN-mediated DNA insertion has not yet been used specifically for PMF applications, but it has been described in a handful of studies in different species including Arabidopsis (Miki et al. 2018), maize (Svitashev et al. 2015), barley (Watanabe et al. 2016), tobacco, rice (Li et al. 2016), soybean (Bonawitz et al. 2019) and potato (Forsyth et al. 2016). These experiments have involved different types of SSNs and delivery methods, and the reported efficiencies of transgene insertion—calculated as number of precise insertion events per 100 transformations—are mostly in the lower single digit range and all rely on the use of strong selectable markers to isolate the desired events. By developing an engineered transgene integration platform based on previously introduced incomplete marker genes at the insertion site, the efficiency of targeted transgene integration has been increased to 41% in tobacco BY-2 cells (Schiermeyer et al. 2019) but its transferability to other cells lines and intact plants remains to be demonstrated. The efficiency of targeted integration is inversely proportional to the size of the DNA fragment, so it becomes more challenging to insert large constructs (> 20 kb) as required for the introduction of multigene constructs for entire metabolic pathways, including the biosynthesis of mammalian-type sialylated *N*-linked glycans. This limitation could be overcome using a SSN-mediated targeted transgene stacking approach in which several transgenes are integrated sequentially in tandem to create a genetically linked molecular stack. This would avoid the cumbersome breeding steps needed to combine different genes in a single plant line.

An experimental setup with two SSN recognition sites flanking the target sequence makes it possible to simultaneously remove one DNA sequence and insert a new one at the same location, a process called transgene replacement (Weinthal et al. 2013; Schneider et al. 2016). This approach could be used to generate final production lines devoid of co-expressed selectable marker genes. First, a generic transgenic ‘acceptor line’ containing a positive/negative selection marker such as *codA* (Shao et al. 2015) at the desired genomic location is generated by homologous recombination (HR) as a means of initial targeted integration. In a subsequent transformation step, the marker gene is replaced with the production transgene, and the lines with successful cassette exchange are recovered by negative selection against *codA*. In the specific case of antibodies, existing high-performance lines could be modified by the targeted replacement of only the variable domains in the antibody transgene.

Ideally, at least one safe-harbor locus should be identified as the site for targeted integration for each plant species and variety used for PMF. This locus would allow sustained, high-level transgene expression because transgene integration would not cause any obvious deleterious phenotypic effects. This would minimize the effort needed to screen for productive lines and would accelerate the characterization and selection of production lines with predictable and consistent performance. Because targeted transgene integration and replacement in higher plants are technically challenging and rather inefficient (Kumar et al. 2016; D’Halluin et al. 2013), more efforts have to be made in understanding how to improve these molecular processes before they can be routinely exploited, also for PMF purposes.

## Improving recombinant protein accumulation

### Codon preferences and tRNA pools

Even if the transcriptional activity of a transgene is maximized by site-directed integration as described above, and gene silencing effects are avoided (see next section), this does not guarantee high-level product accumulation *in planta*, which is the key factor affecting overall process costs, especially when the yield is below 1 g kg^−1^ biomass (Nandi et al. 2016; Fischer and Buyel 2020). In addition to large numbers of mRNAs (Bhullar et al. 2009; Jansing and Buyel 2019) efficient protein synthesis by ribosomes must also be ensured, and this has turned out to be a more complex, multi-parameter problem than expected. The GC content of genes may not only affect transcription due to an influence on chromatin structure (Barahimipour et al. 2015) but can also have an effect on translation as was shown for different untranslated regions and coding sequences (Zhao et al. 2017). Interestingly, others have recently suggested that not GC content (alone) but more importantly codon-anticodon kinetics may be a major driver for translation efficacy (Sahoo et al. 2019).

The phenomenon of codon bias can cause a gene from one (source) organism to be poorly expressed in another (host) due to the prevalence of disfavored codons (Gouy and Gautier 1982; Webster et al. 2017; Mahalik et al. 2014; Liu et al. 2016; Gustafsson et al. 2004). It is possible to identify correlations between the use of specific codons and the resulting quantity of correctly folded and active protein that accumulates in a cell, reflecting the host’s codon preferences. Accordingly, the yield of recombinant protein can be improved by maximizing codon preference (replacing each codon in the mRNA with the preferred codon in the host), or harmonizing codon preference (replacing each codon in the mRNA with an equivalent codon in terms of usage frequency in the host). Even so, mRNA meta-functions such as a 5′ end “speed ramp” of a mRNA, codon autocorrelation and self-folding can interfere with such optimization because features that modulate the rate of translation may be necessary for effective protein synthesis (Tuller et al. 2010; Cannarozzi et al. 2010; Kozak 2001; Jackson et al. 2010). For example, rarer codons at positions corresponding to domain transitions in the protein can assist during folding (Hanson and Coller 2018). When bone morphogenetic protein (BMP) 2 was expressed in tobacco plants, the two-fold accumulation advantage achieved by codon optimization was nullified if a stronger promoter was used (Suo et al. 2006).

The properties discussed above are largely product-centered because they can be addressed by modifying the mRNA sequence. However, the properties of the expression host can also be modified as shown by the augmentation of rare tRNA pools in *Escherichia coli*, which increased the accumulation of proteins relying on these codons (Tegel et al. 2010). Increasing tRNA concentrations alone is unlikely to improve product formation if it merely transfers the bottleneck elsewhere or elicits new challenges. For example, rare codons corresponding to smaller tRNA pools should be able to maintain their function and slow down translation to facilitate folding where appropriate (Hanson and Coller 2018; Webster et al. 2017). Therefore, when translating tRNA pool modifications to PMF applications, it can be prudent to pursue a harmonized approach that maintains the relative abundance of natural tRNA pools rather than using preferred codons throughout the coding sequence.

Codon harmonization requires information about codon usage and the size of tRNA pools in the source organism and the host. The magnitude of tRNA pools is well-characterized in yeast (Bloom-Ackermann et al. 2014; Shah and Gilchrist 2011) but not in plants, although such information may become accessible with recently developed methods, e.g. combined treatment with the demethylating enzyme AlkB and ligation with tRNA-specific adapters in order to sequence tRNAs (Warren et al. 2020). In addition to sequence modifications (Hopper and Nostramo 2019; Hummel et al. 2019), the composition of tRNA pools can change in response to stress (Torrent et al. 2018). Recombinant protein overexpression can induce stress, especially when triggered by bacterial infiltration (Grosse-Holz et al. 2017; Buyel et al. 2015a), and the impact on tRNA pools should therefore be investigated before adapting the expression host. Advances in the detection and quantification of tRNAs can facilitate the rapid and routine analysis of such pools (Jacob et al. 2019) and thus contribute to an enhanced understanding of mRNA translation, specifically of recombinant proteins. Once in place, modified tRNA pools can also help to prevent protein aggregation (Nedialkova and Leidel 2015). Such modifications can be introduced by genome editing, for example by removing or altering the stress response pathway or modifying the expression of tRNA genes.

### Suppression of gene silencing

Sequence-dependent RNA degradation or silencing can be directed against RNA transcribed from transgenes, thereby reducing the yield of recombinant proteins (Brodersen and Voinnet 2006). However, this mechanism can be prevented by the co-expression of viral silencing suppressors. Several of these repressors have been used alone or in combination for transient co-expression in *Nicotiana benthamiana* (Arzola et al. 2011). For example, the p19 suppressor from tomato bushy stunt virus (TBSV) binds to siRNA and prevents RISC assembly, and has been widely used to boost the expression of recombinant proteins, as shown by the 15-fold increase in antibody yields in tobacco plants (Garabagi et al. 2012). Other silencing repressors inhibit local and systemic RNA silencing by preventing the accumulation of siRNAs, interfering with siRNA–AGO interactions, or triggering the degradation of AGO1 (Baumberger et al. 2007). These approaches rely on the overexpression of silencing repressors, but other strategies based on the repression of endogenous genes have been reported. The DCL2 and DCL4 genes were simultaneously repressed in *N. benthamiana* plants by RNA interference (RNAi) to improve the production of recombinant proteins by transient expression, although the repression levels were somewhat unstable (Matsuo and Matsumura 2017). Gene knockouts generated using gene editing technology provide a preferable alternative, and this has been demonstrated in *Medicago truncatula* and soybean by using TALENs and CRISPR/Cas9 to modify DCL2, DCL3 and other genes involved in small RNA processing (Curtin et al. 2018). AGO2 was also inactivated in *N. benthamiana* using CRISPR/Cas9, and infection with a viral vector encoding green fluorescent protein (GFP) resulted in higher expression levels in these plants (Ludman et al. 2017).

The CRISPR/Cas9 system has been used to knock out RNA-dependent RNA polymerase 6 (RDR6) in *N. benthamiana*. This enzyme is required for the synthesis of dsRNAs that are subsequently processed into siRNAs. During transient expression, the resulting plants were defective in post-transcriptional gene silencing and accumulated larger amounts of recombinant GFP than controls (Matsuo and Atsumi 2019). Interestingly, the *N. benthamiana* LAB strain (http://benthgenome.qut.edu.au/) carries a natural frameshift insertion in the *RDR1* gene that affects its response to viral infection and makes it an ideal host for viral expression vectors (Yang et al. 2004). Genome editing may facilitate the transfer of these useful features of the *N. benthamiana* LAB strain to other production hosts, including other *Nicotiana* species (Bally et al. 2018).

### Stress resilience and modified degradation pathways

The expression of secreted recombinant proteins in plants often causes an imbalance between the amount of unfolded protein entering the secretory pathway and the protein folding machinery of the endoplasmic reticulum (ER), resulting in the induction of ER stress and an unfolded protein response (UPR) in which the cell increases its protein-folding capacity (Arcalis et al. 2019; de Wilde et al. 2013; Oono et al. 2010; Wang et al. 2015; Pastor-Cantizano et al. 2020). The major UPR sensing system appears to be conserved in all eukaryotes, although different organisms utilize different subsets of ER-resident transmembrane sensors. When these sensors are activated by unfolded proteins, they initiate a cascade of events that eventually stimulates the synthesis of more chaperones and other enzymes that promote protein folding, such as protein disulfide isomerases (PDIs) that promote the formation of disulfide bonds. The UPR also suppresses the synthesis of secretory proteins to prevent further overloading of the ER (Howell 2013).

Although some protective aspects of the UPR, such as the activation of ERAF (ER-assisted folding) pathways, are likely to encourage the production of functional recombinant proteins, the associated quality control mechanisms, the induction of programmed cell death (PCD), and the general downregulation of secretory protein expression undoubtedly act against protein accumulation. It is therefore necessary to achieve the selective control of specific components of the UPR signaling pathway for the permanent improvement of recombinant protein synthesis in plants. Selective partial UPR activation has also been proposed for mammalian cells and yeast as a means to optimize protein production and secretion (Raschmanová et al. 2019; Hussain et al. 2014). The design of meaningful strategies to this effect requires a thorough understanding of the processes needed to induce the ERAF pathway while avoiding the general suppression of protein synthesis (Thomas and Walmsley 2015).

Inositol requiring enzyme 1 (IRE1) acts as a major signaling hub and comprises an endoribonuclease domain and a kinase domain. Whereas the ribonuclease activity of IRE induces regulated IRE1-dependent decay (RIDD) of mRNAs encoding secretory proteins (Chen and Brandizzi 2013), and would therefore be an obvious target for gene disruption, the complete knockout of IRE1 is detrimental for plant development because the kinase activity of IRE1 plays a key role independent of the ribonuclease activity, as shown in rice using a gene targeting system to replace genomic IRE1 with two types of missense alleles leading to a defect in either the kinase or ribonuclease domain (Wakasa et al. 2012). Today’s genome editing technologies will allow even more efficient targeted modifications, making it feasible to generate plant expression hosts lacking the RNase domain of IRE1 while maintaining its kinase activity.

In addition to modulating individual pathways, it is also important to identify key factors in plants that suppress ER stress responses in order to prevent PCD. This could be achieved by designing loss-of-function genetic screens based on CRISPR/Cas9, as recently demonstrated in mammalian cells (Panganiban et al. 2019).

### Enhancing the protein storage capacity of the endomembrane system

#### The ER—central hub for protein synthesis, folding, modification and storage

Most complex recombinant proteins produced in plants pass through the ER and acquire ER-specific post-translational modifications; some are even retained in this compartment (Margolin et al. 2020). Cells that secrete large amounts of protein tend to feature a well-developed ER that accounts for a substantial proportion of the cellular volume. For example, antibody-secreting mammalian plasma cells differentiate from B lymphocytes, and this process is marked by a substantial expansion of the ER (Zhu et al. 2019). In plants, a similar phenomenon is observed in seed endosperm cells, which produce large quantities of storage proteins (Arcalís et al. 2020). Not surprisingly, a positive correlation has also been established between the effective volume of the ER and the capacity of cells to secrete recombinant proteins, and yeast strains with an expanded ER have been shown to produce higher yields of such proteins (Ruijter et al. 2016). UPR signaling is also linked to ER membrane expansion driven by lipid biosynthesis, and membrane expansion alleviates ER stress independently of an increase in ER chaperone levels. Therefore, expanding the ER by promoting membrane synthesis is not only a means to increase the capacity and productivity of the ER, but also an important component of the cellular mechanism to overcome ER stress (Schuck et al. 2009; Ruijter et al. 2016). In order to increase ER capacity, cells can be engineered to produce larger quantities of phospholipids, especially phosphatidylcholine (PC). One way to boost intracellular PC levels is to reduce or abolish the catalytic activity of a cytosolic phosphatidic acid phosphatase (PAP or PAH), which leads to ER proliferation, as demonstrated by the double knockout of PAH1/2 in Arabidopsis (Craddock et al. 2015). A similar effect could be achieved in other plant species by genome editing.

#### The vacuole—storage opportunity or dead volume?

Plant vacuoles account for up to 90% of the vegetative cell volume and usually function as lytic compartments (Marty 1999). In seeds and other storage organs, vacuolar compartments are often specialized for the long-term storage of stable protein reserves, serving as a protein storage vacuole (PSV) for the stockpiling of nutrients (Herman and Larkins 1999). The PSV is therefore a favorable intracellular destination for recombinant proteins produced in seeds (Takaiwa et al. 2017; Arcalis et al. 2014).

In contrast, the deposition of recombinant proteins in the central vacuole of leaf cells and undifferentiated suspension cells is often considered undesirable because, for many proteins, this compartment does not provide a stable environment. However, there are several examples of recombinant proteins (including avidin, cellulolytic enzymes and endolysin) that accumulate to high levels in the leaf central vacuole (Marin Viegas et al. 2017). Vacuolar targeting has also been reported in *N. benthamiana* for monoclonal antibodies (Ocampo et al. 2016). Most notably, a vacuolar targeting signal was used for the expression of human glucocerebrosidase in carrot cells, the first recombinant protein produced in plants that was approved and marketed as a pharmaceutical product for human use. In this case, vacuolar targeting was used to achieve the desired *N*-linked glycan structure with terminal mannose residues, resulting from the activity of a vacuolar glycan-modifying enzyme (Shaaltiel et al. 2007).

Despite the cases described above, the lytic character of the vacuole is often a disadvantage. However, vacuoles of vegetative tissues are highly dynamic, and their characteristics are affected by environmental conditions, developmental programs and genetic cues, and therefore culture conditions and genetic modulation may be suitable tools for optimization. For example, transient overexpression of the key transcriptional regulator LEAFY COTYLEDON2 (LEC2) alters leaf morphology and the lytic vacuole becomes smaller in size and is replaced by PSVs (Feeney et al. 2013). The controlled transcriptional activation of such regulators may allow the vacuolar compartments to be modified or minimized in the context of PMF. This could be achieved, for example, using the CRISPR activation (CRISPRa) approach, in which a mutant version of Cas9 with both nuclease domains inactivated (known as dead Cas9 or dCas9) but retaining its ability to bind specific DNA sequences when directed by guide RNA (gRNA), is fused to a transcriptional activator domain (Chavez et al. 2015). In the long term, it may be possible to induce the transformation of vacuoles into PSVs, for example during transient expression, but this substantial intervention in cellular metabolism may have negative side effects that first have to be understood and addressed.

### Modulating levels of endogenous chaperones

The folding and maturation of proteins in the ER is mediated by chaperones, which are also involved in the stringent quality control of nascent proteins to ensure that terminally misfolded proteins are targeted for ERAD (Strasser 2018). The production of recombinant proteins frequently imposes stress on this machinery, inducing the UPR and ultimately increasing the expression of chaperones, thus providing additional protein folding capacity. Not surprisingly, this sparked the idea to overexpress selected chaperones in order to support the production of endogenous and recombinant proteins in larger amounts. However, the substantial overexpression or suppression of BiP1 in rice triggered ER stress, altering the seed phenotype and the intracellular structure of the endosperm, thus reducing the seed protein content (Wakasa et al. 2011). Interestingly, the highest recombinant protein yield was achieved in rice seeds expressing slightly higher than normal levels of BiP1, leading to the conclusion that only judicious modification of BiP1 levels and a well-balanced abundance of ER stress-related proteins in transgenic rice would enhance the production of secretory proteins (Wakasa et al. 2011). Similarly, in yeast and mammalian cells, the overexpression of single ERAF genes such as BiP and PDI had variable success in terms of product yields (Klabunde et al. 2007; Damasceno et al. 2007; Kunert and Reinhart 2016). The more subtle modulation of the entire ERAF pathway by targeting and editing key regulators may be necessary for consistent host plant improvement.

An alternative approach is the co-expression of chaperones and folding helpers from the source of the recombinant protein. For example, in the presence of human CRT, several human viral glycoproteins accumulated to much higher levels in *N. benthamiana* compared to hosts with the plant chaperone machinery alone. Furthermore, the host ER stress response induced by HIV Env gp140 expression was alleviated in the presence of human CRT (Margolin et al. 2020). It will be interesting to explore further combinations of recombinant proteins and chaperones from different origins to identify successful strategies for the optimization of host secretory pathways for the production of specific protein classes. Clearly, a combination of genome editing and genetic modification will be required to reach this goal.

### Modulating endogenous protease activity

One of the major hurdles preventing the broader adoption of PMF is the relatively low yield of intact recombinant protein compared to established production platforms. The low yields partly reflect the presence of endogenous proteases, which can degrade the product *in planta*, in the supernatant of cell suspension cultures, or following the disruption of the plant tissue for product extraction. This not only reduces the yields, but can also interfere with DSP and affect product quality because the degradation products are difficult to remove. For example, full-size IgG antibodies, by far the largest class of biopharmaceuticals, frequently suffer from proteolytic degradation when expressed in plants (Donini et al. 2015; Puchol Tarazona et al. 2020). The most straightforward strategy to avoid proteolysis is to deplete or eliminate the native plant proteases at their source. RNAi-mediated gene silencing for the downregulation of certain protease classes can boost the accumulation of recombinant proteins in rice (Kim et al. 2008), tobacco BY-2 cells (Mandal et al. 2014), and tobacco leaves (Duwadi et al. 2015). Although this is a valid strategy, RNAi triggers a more generalized gene silencing response that could affect transgene expression, and is certainly not compatible with transient expression methods that involve the co-expression of silencing inhibitors. RNAi is also sensitive to environmental factors such as warm temperatures, which suppress post-transcriptional gene silencing in Arabidopsis (Zhong et al. 2013). The knockout of protease genes by genome editing is a more effective approach. Different proteases can contribute to the degradation of a target protein by attacking different regions, so multiplex editing would be required to remove all relevant proteases (Schiermeyer 2020). In such cases, the extraordinary versatility of the CRISPR/Cas9 system is a great advantage, particularly its compatibility with multiplex editing. A specific subtilisin family protease was knocked out by gene targeting in the moss *Physcomitrella patens*, leading to a significant reduction in extracellular proteolytic activity and a small increase in recombinant protein yields (Hoernstein et al. 2018). Technically this was achieved by HR, the predominant repair pathway in moss, which occurs spontaneously in the absence of DSBs. In higher plants, where HR is less efficient, the simplest approach to inactivate protease genes would be SSN-induced indel formation.

The engineered *P. patens* line lacking subtilisin appeared phenotypically identical to wild-type moss, but the overall effect of protease knockouts in higher plant development or defense cannot be predicted and would need to be evaluated in each case (van der Hoorn 2008). The use of inducible promoters to achieve the spatiotemporal control of SSN expression would allow the depletion or elimination of proteases to be coupled with the time of production and harvest, thus avoiding any interference with plant growth. Recently, an estrogen-triggered CRISPR/Cas9 system that conditionally generates targeted gene knockouts in particular cell types has been reported in Arabidopsis (Wang et al. 2020). The dCas9 system discussed above could also be used, this time with the inactive SSN fused to a transcriptional repressor, to modulate several protease families simultaneously with only one regulatory protein (Lowder et al. 2017).

Alternatively, Cas13 could be used to downregulate gene expression without triggering silencing. Cas13 is a recent addition to the CRISPR toolbox that can specifically degrade single-stranded RNA, and it is easily reprogrammed to target any sequence of choice. Cas13 has already been used for the post-transcriptional regulation of gene expression in plants (Wolter and Puchta 2018). In any case, when the co-expression of additional proteins is required in PMF applications, preliminary validation must be carried out to ensure there is no impact on the yield of the primary recombinant protein.

There are several hundred proteases with various activities and expression profiles in different plant species and subcellular compartments (van der Hoorn 2008), so there is no easy and one-size-fits-all solution to solve the issue of proteolytic degradation. For each production process and target protein, the genes of relevant proteases should be identified and then knocked out or downregulated. Genome editing offers an excellent research tool to identify individual proteases acting on a given protein, allowing anti-protease strategies to be tailored for different PMF processes. Given the high throughput of the CRISPR/Cas9 system, it is now possible to conduct systematic screens of large numbers of genes, for example to identify protease inhibitors that enhance the accumulation of pharmaceutical proteins in *N. benthamiana* (Grosse-Holz et al. 2018). Such an approach can be particularly useful to identify groups of proteases that are relevant for the degradation of various recombinant protein products, thereby facilitating their knockout and the establishment of a general-purpose expression host plant line.

As an alternative to targeted protease inactivation, the large number of proteases in plants can be addressed by expressing broad-spectrum protease inhibitors (PIs) together with the recombinant protein of interest. PIs can be targeted to the same subcellular compartment as the recombinant protein product, and a single PI is often effective against several proteases with redundant functions (Grosse-Holz and van der Hoorn 2016). However, strict spatiotemporal control of PI expression is important to avoid compromising host plant development. This is easily achieved in transient expression systems based on agroinfiltration because the PI is only expressed in the infiltrated tissues, avoiding unwanted effects elsewhere (Grosse-Holz et al. 2018). For example, the tomato cysteine protease inhibitor SlCYS8 boosted the accumulation of antibodies transiently expressed in *N. benthamiana* leaves (Jutras et al. 2016) and has also been used as a stabilizing fusion partner for other recombinant proteins (Sainsbury et al. 2013). More recently, two new *N. benthamiana* PIs (NbPR4, NbPot1) and one of human origin (HsTIMP) increased the accumulation of α-galactosidase, erythropoietin, and a monoclonal antibody in infiltrated *N. benthamiana* leaves (Grosse-Holz et al. 2018). Other PIs that enhance the accumulation of recombinant proteins have been expressed in transgenic plants or plant cells either constitutively or under the control of inducible or organ-specific regulatory elements (Mandal et al. 2016; Pillay et al. 2012). Genome editing may provide a useful tool to modify the promoters of endogenous PI genes to achieve spatiotemporally regulated overexpression.

## Modifying post-translational modifications and product quality

### Specific *N*-linked and *O*-linked glycosylation profiles

Glycosylation is one of the most important post-translational modifications in the context of PMF. The presence and structure of sugar residues influences protein homogeneity, assembly, immunogenicity and functionality, such as the ability of mAbs to trigger antibody dependent cellular cytotoxicity (Chenoweth et al. 2020). Animals and plants produce complex *N*-linked glycans with an identical core of two *N*-acetylglucosamine (GlcNAc) residues followed by a bifurcating mannose, additional mannose residues on each branch, and terminal GlcNac residues, a structure described by the abbreviation GnGn (Montero-Morales and Steinkellner 2018). There are three main differences between the complex glycans of plants and humans: (1) plant glycans typically carry core α(1,3)fucose and β(1,2)xylose, which are not present in humans; (2) some recombinant proteins produced in plants, including human erythropoietin, are modified by adding β(1,3)galactose and α(1,4)fucose to the terminal GlcNAc residues, forming a structure known as the Lewis a (Le^a^) trisaccharide (Weise et al. 2007; Castilho et al. 2013) which occurs only rarely on the glycoproteins of healthy adult humans (Parsons et al. 2013); (3) paucimannosidic-type glycans lack the two terminal GlcNAc residues, which are trimmed off by specific β-*N*-acetylhexosaminidases (HEXO). These differences prevent the addition of homogeneous human-like galactosylated *N*-linked and *O*-linked glycans on recombinant glycoproteins produced in plants (Kriechbaum et al. 2020).

Because the specific pattern of glycosylation may affect a product’s performance, e.g. effector function, modifying plant-specific glycosylation can be of interest (Nagels et al. 2011). The elimination of plant-specific β(1,2)xylose and α(1,3)fucose residues in *P. patens* was achieved by HR (Koprivova et al. 2004). RNAi and chemical mutagenesis with ethylmethanesulfonate (EMS) have been used to generate duckweed (Cox et al. 2006), alfalfa (Sourrouille et al. 2008) and *N. benthamiana* lines (Strasser et al. 2008) with significantly depleted β(1,2)xylosylated and/or α(1,3)fucosylated glycans, but residual amounts of these structures remain. This is because RNAi does not completely eliminate all target mRNAs, and EMS mutagenesis was unable to simultaneously inactivate multiple fucosyltransferase genes. In contrast, SSNs can induce precise mutations in multiple target genes ensuring complete enzyme inactivation. CRISPR/Cas9 is particularly suitable for multiplex gene editing, allowing the generation of *N. benthamiana* plants (Jansing et al. 2018) and tobacco BY-2 cell suspension cultures (Mercx et al. 2017; Hanania et al. 2017) completely devoid of β(1,2)xylose and α(1,3)fucose by knocking out up to seven genes simultaneously.

Le^a^ epitopes were eliminated in *P. patens* by HR-mediated knockout of the β(1,3)galactosyltransferase gene (Parsons et al. 2012) and it is only a matter of time before the same is achieved using SSNs in higher plants. Recently, the improved β(1,4)galactosylation of proteins other than monoclonal antibodies (Strasser et al. 2009) and at different glycosylation sites was reported in *N. benthamiana* using an RNAi approach to target the β-galactosidase 1 gene (Kriechbaum et al. 2020).

There is also much interest in simplifying the plant glycosylation repertoire to generate a minimal glycan core structure (GnGn) that can be exploited as an acceptor substrate for further elongation and diversification (Montero-Morales and Steinkellner 2018). Building such a platform requires that plant-specific β(1,2)xylose and α(1,3)fucose residues are completely removed and that the hydrolysis of GlcNAc from complex glycans is prevented to provide homogeneous GnGn “acceptor” structures. RNAi targeting of HEXO genes led to the depletion of paucimannosidic *N*-linked glycans in *N. benthamiana* (Shin et al. 2017). Because RNAi only downregulates genes and is sensitive to variable experimental conditions, leading to inconsistent and heterogeneous glycosylation patterns (Kallolimath et al. 2020), it is important to achieve stable gene disruption at the DNA level by genome editing to generate a robust chassis. The full integration of the different steps needed to generate a plant with no plant-specific β(1,2)xylose and α(1,3)fucose and no degradation of terminal GlcNAc has yet to be achieved (Fischer et al. 2018). Once such a chassis is available, the enzymatic machinery for the synthesis, transport and addition of galactosylated and sialylated *N*-linked glycans (Kallolimath et al. 2016) can be introduced by conventional transformation to ultimately produce recombinant proteins with a homogeneous, human-like glycosylation profiles.

A GlycoDelete strategy has been applied in Arabidopsis, for which an EMS mutant lacking *N*-acetylglucosaminyltransferase-I (GnTI) activity is available (Piron et al. 2015). Overexpression of a Golgi-targeted version of a fungal endo-*N*-acetyl-β-d-glucosaminidase in this mutant background resulted in a line producing recombinant proteins with homogeneous, single-GlcNAc *N*-linked glycans and without any obvious phenotype. Such simplified *N*-glycosylation pathways could be useful for the production of human proteins that benefit from mannosidic structures, such as glucocerebrosidase (Limkul et al. 2016), without needing to modify their sequence for retention in specific subcellular compartments. With genome editing technologies, it is now feasible to quickly generate the GnTI mutants and extend the robust GlycoDelete strategy to species other than Arabidopsis.

Despite the importance of *O*-linked glycans for the biological activity and pharmacological properties of recombinant proteins, and the fact that plant-specific *O*-linked glycans are IgE epitopes in allergy patients (Gomord et al. 2010), these structures have received far less attention than *N*-linked glycans. Whereas mammalian proteins predominantly bear mucin-type glycans added to serine and threonine, *O*-linked glycosylation in plants starts with the conversion of proline to hydroxyproline by enzymes of the prolyl-4-hydroxylase (P4H) family, followed by decoration with arabinose or arabinogalactan residues (Strasser 2016). The first step towards humanizing plant *O*-linked glycans is therefore to knockout the P4H genes. This was achieved by HR in *P. patens*, allowing the production of recombinant human erythropoietin devoid of non-human prolyl-hydroxylation and without obvious phenotypic modifications in the host (Parsons et al. 2013). Research is focusing on the identification and elimination of P4H isoforms involved in the synthesis of hydroxyproline in *N. benthamiana* (Schoberer and Strasser 2018). The multiplexing capability of the CRISPR/Cas9 system makes it the most suitable tool to inactivate multiple P4H paralogs.

The introduction of mucin-type *O*-glycosylation in higher plants was achieved by overexpressing the human GalNAc transferase 2 gene in Arabidopsis, tobacco BY-2 cells and *N. benthamiana* (Montero-Morales and Steinkellner 2018). Transient co-expression with constructs encoding enzymes required for the initiation and elongation of human *O*-linked glycans made it possible to generate disialylated mucin-type core 1 *O*-linked glycans on IgA1 produced in ΔXT/FT *N. benthamiana* plants (Dicker et al. 2016). Targeted gene integration mediated by SSNs would ensure co-segregation of the multiple genes required to introduce human-like glycosylation pathways in stable transgenic plants.

### Modulating endogenous oxidase activity

All plants produce a combination of phenolic compounds, which are released during the homogenization step required to extract PMF products, especially from green tissues. Polyphenol oxidases (PPOs) can form covalent complexes between phenols and proteins that can result in protein aggregation and precipitation, substantially reducing the yield, purity and product quality (Twyman et al. 2003).

Phenolic oxidation during extraction from leafy materials can be reduced by supplementing the extraction buffer with antioxidants (Buyel et al. 2015b), but this increases the complexity of the extraction procedure and the overall costs of large-scale production. RNAi targeting the PPO gene has been shown to reduce the browning of potato tubers and apples (Nadakuduti et al. 2018). Accordingly, the elimination or reduction of PPO activity by genome editing would provide an alternative and efficient solution for phenolic oxidation in PMF applications. Knocking out a single member of the potato PPO gene family using CRISPR/Cas9 reduced PPO enzyme activity in the tubers by up to 69% without other phenotypic effects (González et al. 2019), and a similar approach could be used in PMF hosts such as tobacco. Because PPOs play an important role in plant defense, the complete elimination of PPO activity might interfere with normal plant growth, especially during open-field cultivation, and the methods discussed above for spatiotemporal regulation should therefore be considered to avoid negative effects.

## Improved biomass yield and handling

Several parameters can be used to evaluate methods intended to improve biomass yields and handling properties from a biomanufacturing perspective. The most prominent is the harvest index, which was initially defined as the (grain) yield per mass of aboveground dry matter (Hay 1995). This has been adapted for leafy crops and is expressed either as the ratio of leaf biomass to total biomass—the leaf mass fraction (Robson et al. 1996), or the leaf dry mass as a proportion of total plant dry mass (Poorter et al. 2012). Other established metrics for leafy crops include the leaf area index (total leaf area per unit cultivation area) (Pierik et al. 2004), relative growth rate (increase in plant mass per unit plant mass and time), and total dry mass (Poorter et al. 2012). In tobacco, the youngest leaves can accumulate up to tenfold more recombinant protein than older leaves as a proportion of biomass (Buyel and Fischer 2012; Sack et al. 2015), so we recommend that PMF performance measures also accommodate factors such as delayed leaf senescence, protein content and fertilizer consumption. These parameters were used when assessing transgenic 9A4 tobacco (Cherry et al. 1991) and various other crops (Hay 1995). Desirable properties may vary depending on the specific application, process or species, but some generalizations are possible (Table 1). In this section, we will discuss how such properties can be improved by genetic engineering and/or genome editing.

**Table 1:** Features of leafy crops that affect productivity and handling properties for plant molecular farming

Parameter	Unit	Relevance	Indicator for
Harvest index^a^	kg kg^−1^	Plant yield	Relevant biomass
Leaf area index	m^2^ m^−2^	Facility yield	Cultivation density
Relative growth rate	kg kg^−1^ d^−1^	Facility yield	Batch time
Total dry mass	kg plant^−1^	Plant yield	Biomass
Accumulation homogeneity	Log10 (youngest to oldest)	Plant yield	Biomass quality
Protein content^b^	g kg^−1^	Plant yield	Biomass quality

^a^Also referred to as leaf mass fraction

^b^The protein content is often measured in terms of total soluble protein and thus depends on the selected extraction conditions

### Modulating HCP expression to simplify DSP and increase synthesis capacity

The ability of plants and plant cells to produce recombinant proteins can be improved if capacity is diverted from non-product HCPs to the recombinant protein product, at least during certain time periods (e.g., shortly before harvest). Reducing the number or abundance of HCPs can also facilitate subsequent product purification, which otherwise requires complex processing techniques that may not be compatible with all target proteins (Buyel et al. 2016; Opdensteinen et al. 2018), and that increase upfront equipment costs in larger-scale processes (Buyel and Fischer 2014). The steady-state synthesis of HCPs and recombinant proteins in transgenic plants reflects the relative abundance of the corresponding mRNAs (Liu et al. 2016) and their translation rate. Strong constitutive promoters such as the double enhanced Cauliflower mosaic virus 35S promoter or endogenous ubiquitin and actin promoters are used to maximize the rate of transcription (Lessard et al. 2002; Liu and Stewart 2016) and thus generate a large pool of product mRNA for translation. However, substantial cellular resources are still devoted to the synthesis of HCPs, and proteins such as ribulose-1,5-bisphosphate carboxylase/oxygenase (RuBisCO) can account for > 40% of the total soluble protein (TSP) in leaf cells (Buyel 2015). An ideal chassis for PMF would feature the time-controlled shutdown of abundant HCP synthesis using inducible RNAi constructs or dCas9-based transcriptional repressors. In transgenic plants, the RNAi construct or dCas9 fusion would be placed under the control of an inducible promoter, such as the ethanol-inducible *alc* promoter from *Aspergillus nidulans* described in more detail below. If the chassis plant is designed for transient expression, the shutdown constructs could be linked to endogenous promoters that are activated by infiltration with *A. tumefacines* (Grosse-Holz et al. 2017).

The shutdown strategy should focus on the most abundant HCPs involved in photosynthesis and general metabolism but should not target proteins required for protein synthesis, associated energy metabolism or supporting functions such as tRNA re-charging, because the resulting negative impact on protein synthesis would offset any positive effects of increased capacity. RuBisCO is one of the key targets because it is highly abundant in green biomass and expendable at least in the days immediately before harvest, when the plant has already accumulated sufficient energy reserves (Robert et al. 2015). The provision of energy reserves can be re-enforced by genetic engineering, as shown for lipid accumulation in tobacco leaves (Zhou et al. 2020). Some experiments even suggest that a 25% reduction in RuBisCO content can increase biomass accumulation by 10% if the carbon dioxide partial pressure is increased to 120 Pa (Kanno et al. 2017). Other target proteins may be related to photosynthesis, cell growth and cell division, as has been suggested for engineering in *E. coli* (Mahalik et al. 2014). Finally, HCPs related to stress responses are also good targets (Sharma et al. 2020). In such cases, the regulatory proteins of pathogens may be useful to suppress host defense reactions as reported for viruses in various eukaryotic cells (Gale et al. 2000; Urquidi Camacho et al. 2020) and tested for proteins from phytopathogenic bacteria (Buyel et al. 2015a).

For recombinant protein production in seed crops, the concomitant reduction of endogenous storage protein accumulation can lead to a 2–10 fold increase in product yields, possibly because space is made available in the storage organelles due to the absence of endogenous seed proteins and/or because of compensatory mechanisms to maintain total nitrogen and sulfur levels in the seed (Takaiwa 2013; Takaiwa et al. 2017). Furthermore, this reduces competition with the otherwise abundant storage proteins for translation, folding and assembly in the ER. For example, depleting the pool of 13-kDa prolamins in rice endosperm increased the yield of recombinant proteins such as cedar pollen allergen by rebalancing the proteome (Kawakatsu and Takaiwa 2012). Thus far, the suppression of endogenous seed storage proteins in PMF applications has typically been achieved by RNAi (Shigemitsu et al. 2012; Yang et al. 2012; Yuki et al. 2012) or through the use of expression hosts carrying conventionally-induced mutations in storage protein genes (Tada et al. 2003). More recently, CRISPR/Cas9 has been used to target storage protein genes in camelina (Lyzenga et al. 2019), sorghum (Li et al. 2018) and wheat (Sánchez-León et al. 2018). Seed crops depleted for endogenous storage proteins by genome editing could be developed as PMF production hosts because their phenotype is considered stable and they do not contain RNAi constructs, reducing the risk of interference between transgene cassettes.

It is important to note that re-balancing the host cell proteome can also be achieved by technical measures such as exposing plants to methyl jasmonate, as shown for transient expression in *N. benthamiana* (Robert et al. 2015). However, such manufacturing-based manipulations add additional levels of complexity and can increase process variability.

### Modifying existing pathways to avoid toxic metabolites or other disadvantageous molecules

Non-food/feed plants like tobacco reduce the likelihood of product contamination in the food/feed chain (Breyer et al. 2012; Commandeur et al. 2003). However, such plants nevertheless pose a risk because they may produce toxic compounds, such as the alkaloid nicotine in the case of tobacco. The purification steps required for biopharmaceutical products ensure that small molecules and protein-based impurities are removed, so they fall below the limit of detection (Ma et al. 2015). However, laborious techniques based on organic solvents may be necessary to deplete them in technical protein formulations (Fu et al. 2010) or products that rely on minimal processing such as antibacterial proteins (McNulty et al. 2020). Developing a chassis for PMF that is devoid of such potentially toxic compounds is therefore appealing. In tobacco, this goal has been achieved by knocking out both alleles of all six genes coding for berberine bridge enzyme-like (BBL) proteins, which are responsible for the final oxidation step in the synthesis of nicotine (Schachtsiek and Stehle 2019). CRISPR/Cas9 was used for this approach, resulting in a > 99.6% reduction of nicotine levels. However, the modulation of secondary metabolism may cause unwanted side effects if key enzymes are targeted. For example, when a homospermidine synthase was overexpressed in tobacco to reduce spermidine levels, the transgenic plants showed a stunted phenotype (Kaiser et al. 2002). Instead of manipulating enzyme expression directly by gene knockout or overexpression, corresponding transcription factors can be targeted to control metabolite concentrations in a spatiotemporally regulated manner (Hayashi et al. 2020).

The unattractive odor of residual plant biomass can prevent subsequent use, for example as building materials (Buyel 2018). Accordingly, modifying a host plant so that odorous substances are not formed even after primary product extraction can increase the compatibility of the residual biomass with integrated processing, and thus improve the economic viability of the overall process. Thus far, research has focused on the introduction of enzymes that enhance the production of aromatics, for example by overexpressing a monoterpene synthase in tobacco to increase limonene levels (Lücker et al. 2004) and thus alter the smell of the plants (El Tamer et al. 2003). PMF applications could be facilitated by introducing enzymes that degrade odorous volatile organic compounds (VOCs) (Hammerbacher et al. 2019; ul Hassan et al. 2015; Agapiou et al. 2016), for example terpenes released during harvest and biomass decomposition (Müller et al. 2004; Schiavon et al. 2017), eliminating the odor of residual biomass and increasing consumer acceptance if the bagasse is used as a byproduct (see below). Genome editing could also be used to terminate metabolic pathways at a point where interference with other relevant metabolites is limited. Importantly, cultivation conditions can also affect the metabolic profile of plants, so the ideal outcome may reflect a balance between technical and biotechnological approaches (Matros et al. 2006; Buyel et al. 2015a).

### Increased biomass accumulation

The yield of harvested biomass can be increased by partitioning more assimilated carbon into the harvested, product-containing tissue (see below) or by using enhanced agronomy practices and crop protection to realize the full genetic yield potential of a plant. The efficiency of photosynthesis can be engineered to increase the net conversion of visible solar energy into biomass, and genes that can be targeted to improve the photosynthetic efficiency of C3 crops have been identified and comprehensively reviewed elsewhere (Long et al. 2015). Efforts thus far to improve photosynthetic efficiency and carbon gain have mainly involved the conventional transformation of plants to modulate endogenous genes or to introduce synthetic pathways (Kromdijk et al. 2016; Głowacka et al. 2018; South et al. 2019). Genome editing will expand this toolbox, providing more precise methods for mutation, transgene integration and the manipulation of regulatory sequences to boost or modulate the expression of endogenous genes. The resulting plants would be useful PMF hosts because although plants show remarkable metabolic flexibility to accommodate high levels of recombinant protein (Schmidt et al. 2019b) the demand for protein synthesis capacity can compromise growth and biomass production (Oey et al. 2009). On the other hand, although the speed and extent of biomass accumulation is an important factor for PMF applications, improvements to energy conversion, carbon assimilation and growth must not affect the yield of recombinant protein per unit of biomass. In this context, concentrating the recombinant protein within a specific fraction of biomass that has been targeted for preferred carbon partitioning, such as the seeds, can achieve the simultaneous goals of higher biomass accumulation and higher product yields (Takaiwa et al. 2017).

### Modification of plant habits to increase space–time yield and safety

The shape and stature of plants not only affects biomass accumulation but can also be adapted to facilitate bioprocessing. For example, stunted growth can increase the volumetric productivity of vertical farms, and a high leaf-to-stem mass ratio can limit the processing of biomass with a low product content, as reported for tobacco (Buyel and Fischer 2012). These properties can be modified to a limited extent by controlling the cultivation conditions, especially lighting (Poorter et al. 2012). However, the optimal conditions are species-dependent (Park and Runkle 2018), which increases process development costs and necessitates the inclusion of more sophisticated equipment in production facilities, such as wavelength-adjustable LED modules or inter-lighting (Tewolde et al. 2018). This increases the upfront infrastructure costs and adds new layers of process complexity that need to be calibrated, documented and maintained. Furthermore, controlling plant stature by modulating light and other cultivation conditions can have unintended side effects, such as influencing the production of secondary metabolites that interfere with DSP (Buyel et al. 2015a; Darko et al. 2014).

Genetic modifications can also be used to control plant shape and stature, thus avoiding the need for additional technical installations. For example, the *Rht1* and *Rht2* genes control the wheat dwarfing phenotype responsible for ~ 60% of the increased grain yield during the Green Revolution in the 1960s (Khush 2001). However, such phenotypes may not be ideal for leafy crops such as tobacco because they reduce the overall plant biomass (Langridge 2014). Alternatively, overexpressing recombinant proteins can inhibit stem elongation. For example, transgenic tobacco line 9A4 produces an oat phytochrome protein, and is 80% shorter than wild-type controls (Cherry et al. 1991). Phytochromes can trigger additional desirable effects (stress tolerance and fewer lateral branches) as well as unwanted phenotypes (precocious seed germination) as reported in tomato (Ganesan et al. 2017). Genes such as *PHYA* and *PHYB* have been identified as prime targets for modifying plant stature, and a detailed analysis of phytochromes in tobacco has shown that low levels of phyB1 reduce leaf size whereas low levels of phyB2 increase stem length (Fragoso et al. 2017). However, intra-batch variability may increase and plants can become more sensitive to environmental factors such as light (Pierik et al. 2004; Robson et al. 1996). Because it can be difficult to fine tune transgene expression, a gene editing approach may be more prudent than genetic engineering (Buyel et al. 2013).

Genes controlling flowering and senescence can also improve the properties of PMF hosts by influencing stem elongation and biomass quality. For example, CRISPR/Cas9 was used to inactivate the tobacco *FT5* gene, which encodes a floral activator, allowing flowering in a short-day setting but preventing it under long-day conditions (Schmidt et al. 2019a). Therefore, homozygous *ft5*^–^ plants would remain in the vegetative state indefinitely and continue to accumulate biomass under long-day conditions, providing twin advantages for PMF applications: high biomass production and an enhanced biosafety profiles due to the absence of pollen and seed dispersal. Similar results were achieved when three other *FT* genes (*FT1*, *FT2* and *FT3*) were overexpressed in tobacco because the corresponding proteins are floral repressors, causing the plants to remain in the vegetative growth phase under long-day conditions (Harig et al. 2012).

Even though many aspects of the genetic regulation of plant growth are still poorly understood (Fankhauser and Christie 2015), there are large databases of genes and corresponding phenotypes available for several plants including tobacco (Lein et al. 2008), which can be used to identify further genes suitable for gene editing.

### Self-catalyzed processing of residual biomass

Regardless of the product type and even if the product accumulates to very high levels (> 5 g kg^−1^ biomass) there will be residual plant biomass that often accounts for > 90% of the total plant mass (Buyel 2018). Using this mass and the substances within it as a cascade biorefinery to generate additional products can improve the overall economic viability of a PMF process. This can be facilitated by endowing plants with the ability to self-catalyze the initial preprocessing and processing steps, including biopolymer degradation and, as stated above, the removal of VOCs that generate unpleasant odors. This can be achieved by expressing the corresponding enzymes alongside the primary PMF product.

The most abundant molecules in residual plant biomass are carbohydrates, including cellulose, which accounts for ~ 30% of the solid matter in tobacco (Sheen 1983). Cellulose is degraded by exoglucanases and endoglucanases to form oligosaccharides and monosaccharides (Bornscheuer et al. 2014). This process is supported by proteins such as expansins, which loosen the cell wall and improve accessibility (Yoon et al. 2016). Biomass-modifying proteins can be produced in large quantities in transgenic plants (Brandon and Scheller 2020), even in the open field (Schmidt et al. 2019b). Plants can be more suitable for the production of exocellulases than microorganisms due to the higher enzyme activity (Klinger et al. 2015). Endoglucanases can be secreted to the apoplast (Xiao et al. 2018), but targeting to the ER (Klose et al. 2015) or plastids (Schmidt et al. 2019b; Fumagalli et al. 2019) helps to prevent the enzymes degrading essential cellulose structures before plants are harvested and homogenized for product extraction. Alternatively, enzyme expression can be restricted to specific organs, such as maize kernels (Vicuna Requesens et al. 2019), or can be induced at a particular time, such as just before harvest, thus reducing the fiber content and rigidity, which can facilitate primary product extraction. Methods to analyze the spatiotemporal activity of regulatory elements in plants are well-established, allowing the most appropriate promoters to be selected (Xiong et al. 2016). For example, the ethanol-inducible *alc* promoter has been used to induce the expression of recombinant enzymes in tobacco (Salter et al. 1998) and other plants (Roslan et al. 2001), and can even be used to restrict expression to specific plant tissues (Schaarschmidt et al. 2004). The method is compatible with large-scale applications because ethanol vapor can be used for induction (Sweetman et al. 2002). This inducible promoter system has been used successfully to mitigate the adverse of effects of cellulase expression in tobacco caused by a constitutive promoter (Klose et al. 2013). Enzymes can also be expressed transiently to avoid any impact during biomass accumulation, as shown for glucanases expressed in *N. benthamiana* using a pepper mottle virus vector (Song and Ryu 2017). However, genome editing can now be used to modify endogenous cellulase promoters directly, allowing strict spatiotemporal expression.

Whole fibers from PMF processes can also be used to manufacture by-products, including building materials (Revuelta-Aramburu et al. 2020). As discussed above, the removal of odors and potentially harmful metabolites from such materials will increase their acceptability. Instead of laborious technical processes such as those used in the juice industry (Iyer et al. 2010) the same outcome can be achieved by expressing enzymes in the plant biomass, as shown by the removal of odorous compounds from garlic (Mirondo and Barringer 2016) and the removal of alkaloids from tobacco (Lin et al. 2016). Genome editing could also be used to prevent the transformation of precursors into odorous compounds by inactivating or removing the corresponding enzymes.

It is likely that successful biomass processing will require the activity of more than one enzyme, as discussed for the conversion of lignocellulosic biomass into sugars (Adsul et al. 2020). Enzymes suitable for such reactions have already been found in plants (Huang et al. 2019), mesophilic microorganisms (Jacomini et al. 2020), and thermophiles (Han et al. 2020), and have been identified via the metagenomic analysis of relevant microbial consortia, such as those found in biogas facilities (Klippel et al. 2019).

## Outlook

In this review, we discuss the properties of plants at the molecular, cellular and organism levels that are most relevant for PMF applications, and highlighted the complementary roles of genetic engineering and genome editing to address remaining challenges. Genome editing has the potential to alleviate many of the shortcomings of earlier genetic manipulation methods because it potentially facilitates the precise rather than random modification of genomes and allows the direct modulation of genes rather than the incomplete or variable outcomes of methods such as RNAi (Table 1). Targeted transgene integration at a safe-harbor locus in plants could also represent a groundbreaking advance from the regulatory perspective. Independently-derived transgenic lines are currently not directly comparable due to position effects and copy number variation, and regulators therefore consider every transgenic plant line as a completely different event that must be evaluated separately. If the site of DNA integration is known and the transgene itself is the only new aspect of the transgenic line, this may reduce the regulatory burden for new transgenic production lines and substantially accelerate their approval and utilization. Additional work is necessary to understand the physiological mechanisms of growth and secondary metabolism that interact with PMF applications, allowing us to translate this knowledge into an ideal chassis for the production of recombinant proteins.. Ultimately, the choice between technical and biotechnological approaches for PMF should integrate multiple factors, including long-term development efforts, operational costs, process reproducibility, product safety, regulatory approval and customer acceptance.

## Abbreviations

DSB: Double strand break
DSP: Downstream processing
EMS: Ethylmethanesulfonate
ERAD: Endoplasmic reticulum-associated degradation
HCP: Host cell protein
PMF: Plant molecular farming
PPO: Polyphenol oxidase
PTM: Post-translational modification
RuBisCO: Ribulose-1,5-bisphosphate carboxylase/oxygenase
SSN: Site-specific nuclease
TSP: Total soluble protein

## References

[CR1] Adsul M, Sandhu SK, Singhania RR, Gupta R, Puri SK, Mathur A (2020). Designing a cellulolytic enzyme cocktail for the efficient and economical conversion of lignocellulosic biomass to biofuels. Enzyme Microb Technol.

[CR2] Agapiou A, Vamvakari JP, Andrianopoulos A, Pappa A (2016). Volatile emissions during storing of green food waste under different aeration conditions. Environ Sci Pollut Res Int.

[CR3] Alam A, Jiang L, Kittleson GA, Steadman KD, Nandi S, Fuqua JL (2018). Technoeconomic modeling of plant-based griffithsin manufacturing. Front Bioeng Biotechnol.

[CR4] Arcalis E, Ibl V, Peters J, Melnik S, Stoger E (2014). The dynamic behavior of storage organelles in developing cereal seeds and its impact on the production of recombinant proteins. Front Plant Sci.

[CR5] Arcalis E, Ibl V, Hilscher J, Rademacher T, Avesani L, Morandini F (2019). Russell-like bodies in plant seeds share common features with prolamin bodies and occur upon recombinant protein production. Front Plant Sci.

[CR6] Arcalís E, Hörmann-Dietrich U, Zeh L, Stoger E (2020). 3D Electron microscopy gives a clue: maize zein bodies bud from central areas of ER sheets. Front Plant Sci.

[CR7] Arzola L, Chen JX, Rattanaporn K, Maclean JM, McDonald KA (2011). Transient co-expression of post-transcriptional gene silencing suppressors for increased in planta expression of a recombinant anthrax receptor fusion protein. Int J Mol Sci.

[CR8] Bally J, Jung H, Mortimer C, Naim F, Philips JG, Hellens R (2018). The rise and rise of *Nicotiana benthamiana*: a plant for all reasons. Annu Rev Phytopathol.

[CR9] Barahimipour R, Strenkert D, Neupert J, Schroda M, Merchant SS, Bock R (2015). Dissecting the contributions of GC content and codon usage to gene expression in the model alga *Chlamydomonas reinhardtii*. Plant J Cell Mol Biol.

[CR10] Baumberger N, Tsai C-H, Lie M, Havecker E, Baulcombe DC (2007). The Polerovirus silencing suppressor P0 targets ARGONAUTE proteins for degradation. Curr Biol CB.

[CR11] Bhullar S, Chakravarthy S, Pental D, Burma PK (2009). Analysis of promoter activity in transgenic plants by normalizing expression with a reference gene. Anomalies due to the influence of the test promoter on the reference promoter. J Biosci.

[CR12] Bloom-Ackermann Z, Navon S, Gingold H, Towers R, Pilpel Y, Dahan O (2014). A comprehensive tRNA deletion library unravels the genetic architecture of the tRNA pool. PLoS Genet.

[CR13] Bonawitz ND, Ainley WM, Itaya A, Chennareddy SR, Cicak T, Effinger K (2019). Zinc finger nuclease-mediated targeting of multiple transgenes to an endogenous soybean genomic locus via non-homologous end joining. Plant Biotechnol J.

[CR14] Bornscheuer U, Buchholz K, Seibel J (2014). Enzymatic degradation of (ligno) cellulose. Angew Chem Int Ed Engl.

[CR15] Brandon AG, Scheller HV (2020). Engineering of bioenergy crops: dominant genetic approaches to improve polysaccharide properties and composition in biomass. Front Plant Sci.

[CR16] Breyer D, de Schrijver A, Goossens M, Pauwels K, Herman P, Wang A, Ma S (2012). Biosafety of molecular farming in genetically modified plants. Molecular farming in plants. Recent advances and future prospects.

[CR17] Brodersen P, Voinnet O (2006). The diversity of RNA silencing pathways in plants. Trends Genet TIG.

[CR18] Buyel JF (2015). Process development strategies in plant molecular farming. Curr Pharm Biotechnol.

[CR19] Buyel JF (2018). Plant molecular farming—integration and exploitation of side streams to achieve sustainable biomanufacturing. Front Plant Sci.

[CR20] Buyel JF, Fischer R (2012). Predictive models for transient protein expression in tobacco (*Nicotiana tabacum* L.) can optimize process time, yield, and downstream costs. Biotechnol Bioeng.

[CR21] Buyel JF, Fischer R (2014). Generic chromatography-based purification strategies accelerate the development of downstream processes for biopharmaceutical proteins produced in plants. Biotechnol J.

[CR22] Buyel JF, Kaever T, Buyel JJ, Fischer R (2013). Predictive models for the accumulation of a fluorescent marker protein in tobacco leaves according to the promoter/5′UTR combination. Biotechnol Bioeng.

[CR23] Buyel JF, Buyel JJ, Haase C, Fischer R (2015). The impact of *Pseudomonas syringae* type III effectors on transient protein expression in tobacco. Plant Biol (Stuttgart).

[CR24] Buyel JF, Twyman RM, Fischer R (2015). Extraction and downstream processing of plant-derived recombinant proteins. Biotechnol Adv.

[CR25] Buyel JF, Hubbuch J, Fischer R (2016). Comparison of tobacco host cell protein removal methods by blanching intact plants or by heat treatment of extracts. Jove J Vis Exp.

[CR26] Cannarozzi G, Cannarrozzi G, Schraudolph NN, Faty M, von Rohr P, Friberg MT (2010). A role for codon order in translation dynamics. Cell.

[CR27] Castilho A, Neumann L, Gattinger P, Strasser R, Vorauer-Uhl K, Sterovsky T (2013). Generation of biologically active multi-sialylated recombinant human EPOFc in plants. PLoS ONE.

[CR28] Chavez A, Scheiman J, Vora S, Pruitt BW, Tuttle M, Iyer EPR (2015). Highly efficient Cas9-mediated transcriptional programming. Nat Methods.

[CR29] Chen Y, Brandizzi F (2013). IRE1: ER stress sensor and cell fate executor. Trends Cell Biol.

[CR30] Chenoweth AM, Wines BD, Anania JC, Mark Hogarth P (2020). Harnessing the immune system via FcγR function in immune therapy: a pathway to next-gen mAbs. Immunol Cell Biol.

[CR31] Cherry JR, Hershey HP, Vierstra RD (1991). Characterization of tobacco expressing functional oat phytochrome: domains responsible for the rapid degradation of Pfr are conserved between monocots and dicots. Plant Physiol.

[CR32] Commandeur U, Twyman RM, Fischer R (2003). The biosafety of molecular farming in plants. AgBiotechNet.

[CR33] Cox KM, Sterling JD, Regan JT, Gasdaska JR, Frantz KK, Peele CG (2006). Glycan optimization of a human monoclonal antibody in the aquatic plant Lemna minor. Nat Biotechnol.

[CR34] Craddock CP, Adams N, Bryant FM, Kurup S, Eastmond PJ (2015). Regulation of endomembrane biogenesis in arabidopsis by phospatidic acid hydrolase. Plant Signal Behav.

[CR35] Curtin SJ, Xiong Y, Michno J-M, Campbell BW, Stec AO, Čermák T (2018). CRISPR/Cas9 and TALENs generate heritable mutations for genes involved in small RNA processing of *Glycine max* and *Medicago truncatula*. Plant Biotechnol J.

[CR36] Damasceno LM, Anderson KA, Ritter G, Cregg JM, Old LJ, Batt CA (2007). Cooverexpression of chaperones for enhanced secretion of a single-chain antibody fragment in *Pichia pastoris*. Appl Microbiol Biotechnol.

[CR37] Darko E, Heydarizadeh P, Schoefs B, Sabzalian MR (2014). Photosynthesis under artificial light: the shift in primary and secondary metabolism. Philos Trans R Soc Lond Ser B Biol Sci.

[CR38] de Ruijter JC, Koskela EV, Frey AD (2016). Enhancing antibody folding and secretion by tailoring the *Saccharomyces cerevisiae* endoplasmic reticulum. Microb Cell Factories.

[CR39] de Wilde K, de Buck S, Vanneste K, Depicker A (2013). Recombinant antibody production in Arabidopsis seeds triggers an unfolded protein response. Plant Physiol.

[CR300] D’Halluin K, Vanderstraeten C, van Hulle J, Rosolowska J, van den Brande I, Pennewaert A, D'Hont K, Bossut M, Jantz D, Ruiter R, Broadhvest J (2013). Targeted molecular trait stacking in cotton through targeted double-strand break induction. Plant Biotechnol J.

[CR40] Dicker M, Tschofen M, Maresch D, König J, Juarez P, Orzaez D (2016). Transient glyco-engineering to produce recombinant IgA1 with defined *N*- and *O*-glycans in plants. Front Plant Sci.

[CR41] Donini M, Lombardi R, Lonoce C, Di Carli M, Marusic C, Morea V, Di Micco P (2015). Antibody proteolysis: a common picture emerging from plants. Bioengineered.

[CR42] Doudna JA, Charpentier E (2014). Genome editing. The new frontier of genome engineering with CRISPR-Cas9. Science (New York, N.Y.).

[CR43] Duwadi K, Chen L, Menassa R, Dhaubhadel S (2015). Identification, characterization and down-regulation of cysteine protease genes in tobacco for use in recombinant protein production. PLoS ONE.

[CR44] El Tamer MK, Smeets M, Holthuysen N, Lücker J, Tang A, Roozen J (2003). The influence of monoterpene synthase transformation on the odour of tobacco. J Biotechnol.

[CR45] Fankhauser C, Christie JM (2015). Plant phototropic growth. Curr Biol CB.

[CR46] Feeney M, Frigerio L, Cui Y, Menassa R (2013). Following vegetative to embryonic cellular changes in leaves of Arabidopsis overexpressing LEAFY COTYLEDON2. Plant Physiol.

[CR47] Fischer R, Buyel JF (2020). Molecular farming—the slope of enlightenment. Biotechnol Adv.

[CR48] Fischer R, Schillberg S, Hellwig S, Twyman RM, Drossard J (2012). GMP issues for recombinant plant-derived pharmaceutical proteins. Biotechnol Adv.

[CR49] Fischer R, Holland T, Sack M, Schillberg S, Stoger E, Twyman RM, Buyel JF (2018). Glyco-engineering of plant-based expression systems. Adv Biochem Eng Biot.

[CR50] Forsyth A, Weeks T, Richael C, Duan H (2016). Transcription activator-like effector nucleases (TALEN)-mediated targeted DNA insertion in potato plants. Front Plant Sci.

[CR51] Fragoso V, Oh Y, Kim S-G, Gase K, Baldwin IT (2017). Functional specialization of *Nicotiana attenuata* phytochromes in leaf development and flowering time. J Integr Plant Biol.

[CR52] Fu H, Machado PA, Hahm TS, Kratochvil RJ, Wei CI, Lo YM (2010). Recovery of nicotine-free proteins from tobacco leaves using phosphate buffer system under controlled conditions. Bioresour Technol.

[CR53] Fumagalli M, Gerace D, Faè M, Iadarola P, Leelavathi S, Reddy VS, Cella R (2019). Molecular, biochemical, and proteomic analyses of transplastomic tobacco plants expressing an endoglucanase support chloroplast-based molecular farming for industrial scale production of enzymes. Appl Microbiol Biotechnol.

[CR54] Gale M, Tan SL, Katze MG (2000). Translational control of viral gene expression in eukaryotes. Microbiol Mol Biol Rev.

[CR55] Ganesan M, Lee H-Y, Kim J-I, Song P-S (2017). Development of transgenic crops based on photo-biotechnology. Plant Cell Environ.

[CR56] Garabagi F, Gilbert E, Loos A, McLean MD, Hall JC (2012). Utility of the P19 suppressor of gene-silencing protein for production of therapeutic antibodies in *Nicotiana* expression hosts. Plant Biotechnol J.

[CR57] Głowacka K, Kromdijk J, Kucera K, Xie J, Cavanagh AP, Leonelli L (2018). Photosystem II Subunit S overexpression increases the efficiency of water use in a field-grown crop. Nature Commun.

[CR58] Gomord V, Fitchette A-C, Menu-Bouaouiche L, Saint-Jore-Dupas C, Plasson C, Michaud D, Faye L (2010). Plant-specific glycosylation patterns in the context of therapeutic protein production. Plant Biotechnol J.

[CR59] González MN, Massa GA, Andersson M, Turesson H, Olsson N, Fält A-S (2019). Reduced enzymatic browning in potato tubers by specific editing of a polyphenol oxidase gene via ribonucleoprotein complexes delivery of the CRISPR/Cas9 system. Front Plant Sci.

[CR60] Gouy M, Gautier C (1982). Codon usage in bacteria: correlation with gene expressivity. Nucleic Acids Res.

[CR61] Green C, Tibbetts C (1980). Targeted deletions of sequences from closed circular DNA. Proc Natl Acad Sci U S A.

[CR62] Grosse-Holz FM, van der Hoorn RAL (2016). Juggling jobs: roles and mechanisms of multifunctional protease inhibitors in plants. New Phytol.

[CR63] Grosse-Holz F, Kelly S, Blaskowski S, Kaschani F, Kaiser M, van der Hoorn RAL (2017). The transcriptome, extracellular proteome and active secretome of agroinfiltrated *Nicotiana benthamiana* uncover a large, diverse protease repertoire. Plant Biotechnol J.

[CR64] Grosse-Holz F, Madeira L, Zahid MA, Songer M, Kourelis J, Fesenko M (2018). Three unrelated protease inhibitors enhance accumulation of pharmaceutical recombinant proteins in *Nicotiana benthamiana*. Plant Biotechnol J.

[CR65] Gustafsson C, Govindarajan S, Minshull J (2004). Codon bias and heterologous protein expression. Trends Biotechnol.

[CR66] Hammerbacher A, Coutinho TA, Gershenzon J (2019). Roles of plant volatiles in defence against microbial pathogens and microbial exploitation of volatiles. Plant Cell Environ.

[CR67] Han C, Yang R, Sun Y, Liu M, Zhou L, Li D (2020). Identification and characterization of a novel hyperthermostable bifunctional cellobiohydrolase–xylanase enzyme for synergistic effect with commercial cellulase on pretreated wheat straw degradation. Front Bioeng Biotechnol.

[CR68] Hanania U, Ariel T, Tekoah Y, Fux L, Sheva M, Gubbay Y (2017). Establishment of a tobacco BY2 cell line devoid of plant-specific xylose and fucose as a platform for the production of biotherapeutic proteins. Plant Biotechnol J.

[CR69] Hanson G, Coller J (2018). Codon optimality, bias and usage in translation and mRNA decay. Nat Rev Mol Cell Biol.

[CR70] Harig L, Beinecke FA, Oltmanns J, Muth J, Müller O, Rüping B (2012). Proteins from the FLOWERING LOCUS T-like subclade of the PEBP family act antagonistically to regulate floral initiation in tobacco. Plant J Cell Mol Biol.

[CR71] Hay RKM (1995). Harvest index. A review of its use in plant breeding and crop physiology. Ann Appl Biol.

[CR72] Hayashi S, Watanabe M, Kobayashi M, Tohge T, Hashimoto T, Shoji T (2020). Genetic manipulation of transcriptional regulators alters nicotine biosynthesis in tobacco. Plant Cell Physiol.

[CR73] Herman EM, Larkins BA (1999). Protein storage bodies and vacuoles. Plant Cell.

[CR74] Hiatt A, Cafferkey R, Bowdish K (1989). Production of antibodies in transgenic plants. Nature.

[CR75] Hoernstein SNW, Fode B, Wiedemann G, Lang D, Niederkrüger H, Berg B (2018). Host cell proteome of *Physcomitrella* patens harbors proteases and protease inhibitors under bioproduction conditions. J Proteome Res.

[CR76] Hopper AK, Nostramo RT (2019). tRNA Processing and subcellular trafficking proteins multitask in pathways for other RNAs. Front Genet.

[CR77] Howell SH (2013). Endoplasmic reticulum stress responses in plants. Annu Rev Plant Biol.

[CR78] Huang J, Xia T, Li G, Li X, Li Y, Wang Y (2019). Overproduction of native endo-β-1,4-glucanases leads to largely enhanced biomass saccharification and bioethanol production by specific modification of cellulose features in transgenic rice. Biotechnol Biofuels.

[CR79] Hummel G, Warren J, Drouard L (2019). The multi-faceted regulation of nuclear tRNA gene transcription. IUBMB Life.

[CR80] Hussain H, Maldonado-Agurto R, Dickson AJ (2014). The endoplasmic reticulum and unfolded protein response in the control of mammalian recombinant protein production. Biotechnol Lett.

[CR81] Iyer MM, Sacks GL, Padilla-Zakour OI (2010). Impact of harvesting and processing conditions on green leaf volatile development and phenolics in Concord grape juice. J Food Sci.

[CR82] Jackson RJ, Hellen CUT, Pestova TV (2010). The mechanism of eukaryotic translation initiation and principles of its regulation. Nat Rev Mol Cell BioL.

[CR83] Jacob D, Thüring K, Galliot A, Marchand V, Galvanin A, Ciftci A (2019). Absolute quantification of noncoding RNA by microscale thermophoresis. Angew Chem Int Ed Engl.

[CR84] Jacomini D, Bussler L, Corrêa JM, Kadowaki MK, Maller A, da Conceição Silva JL, Simão RDCG (2020). Cloning, expression and characterization of *C. crescentus* xynA2 gene and application of Xylanase II in the deconstruction of plant biomass. Mol Biol Rep.

[CR85] Jansing J, Buyel JF (2019). The correlation between DsRed mRNA levels and transient DsRed protein expression in plants depends on leaf age and the 5′ untranslated region. Biotechnol J.

[CR86] Jansing J, Sack M, Augustine SM, Fischer R, Bortesi L (2018). CRISPR/Cas9-mediated knockout of six glycosyltransferase genes in *Nicotiana benthamiana* for the production of recombinant proteins lacking beta-1,2-xylose and core alpha-1,3-fucose. Plant Biotechnol J.

[CR87] Jutras PV, Marusic C, Lonoce C, Deflers C, Goulet MC, Benvenuto E (2016). An accessory protease inhibitor to increase the yield and quality of a tumour-targeting mAb in *Nicotiana benthamiana* leaves. PLoS ONE.

[CR88] Kaiser A, Sell S, Hehl R (2002). Heterologous expression of a bacterial homospermidine synthase gene in transgenic tobacco: effects on the polyamine pathway. Arch Pharm Pharm Med Chem.

[CR89] Kallolimath S, Castilho A, Strasser R, Grünwald-Gruber C, Altmann F, Strubl S (2016). Engineering of complex protein sialylation in plants. Proc Natl Acad Sci U S A.

[CR90] Kallolimath S, Hackl T, Gahn R, Grünwald-Gruber C, Zich W, Kogelmann B (2020). Expression profiling and glycan engineering of IgG subclass 1–4 in *Nicotiana benthamiana*. Front Bioeng Biotechnol.

[CR91] Kanno K, Suzuki Y, Makino A (2017). A small decrease in Rubisco content by individual suppression of RBCS genes leads to improvement of photosynthesis and greater biomass production in rice under conditions of elevated CO_2_. Plant Cell Physiol.

[CR92] Kawakatsu T, Takaiwa F (2012). Reduction of 13 kD prolamins increases recombinant protein yield and recovery rate in rice endosperm. Plant Signal Behav.

[CR93] Khush GS (2001). Green revolution: the way forward. Nat Rev Genet.

[CR94] Kim N-S, Kim T-G, Kim O-H, Ko E-M, Jang Y-S, Jung E-S (2008). Improvement of recombinant hGM-CSF production by suppression of cysteine proteinase gene expression using RNA interference in a transgenic rice culture. Plant Mol Biol.

[CR95] Klabunde J, Kleebank S, Piontek M, Hollenberg CP, Hellwig S, Degelmann A (2007). Increase of calnexin gene dosage boosts the secretion of heterologous proteins by *Hansenula polymorpha*. FEMS Yeast Res.

[CR96] Klinger J, Fischer R, Commandeur U (2015). Comparison of *Thermobifida fusca* cellulases expressed in *Escherichia coli* and *Nicotiana tabacum* indicates advantages of the plant system for the expression of bacterial cellulases. Front Plant Sci.

[CR97] Klippel B, Blank S, Janzer V-A, Piascheck H, Moccand C, Bel-Rhlid R, Antranikian G (2019). Characterization of a thermoactive endoglucanase isolated from a biogas plant metagenome. Extremophiles Life Under Extreme Cond.

[CR98] Klose H, Gunl M, Usadel B, Fischer R, Commandeur U (2013). Ethanol inducible expression of a mesophilic cellulase avoids adverse effects on plant development. Biotechnol Biofuels.

[CR99] Klose H, Gunl M, Usadel B, Fischer R, Commandeur U (2015). Cell wall modification in tobacco by differential targeting of recombinant endoglucanase from *Trichoderma reesei*. BMC Plant Biol.

[CR100] Knödler M, Rühl C, Emonts J, Buyel JF (2019). Seasonal weather changes affect the yield and quality of recombinant proteins produced in transgenic tobacco plants in a greenhouse setting. Front Plant Sci.

[CR101] Koprivova A, Stemmer C, Altmann F, Hoffmann A, Kopriva S, Gorr G (2004). Targeted knockouts of Physcomitrella lacking plantspecific immunogenic *N*-glycans. Plant Biotechnol J.

[CR102] Kozak M (2001). Constraints on reinitiation of translation in mammals. Nucleic Acids Res.

[CR103] Kriechbaum R, Ziaee E, Grünwald-Gruber C, Buscaill P, van der Hoorn RAL, Castilho A (2020). BGAL1 depletion boosts the level of β-galactosylation of *N*- and *O*-glycans in *N. benthamiana*. Plant Biotechnol J.

[CR104] Kromdijk J, Głowacka K, Leonelli L, Gabilly ST, Iwai M, Niyogi KK, Long SP (2016). Improving photosynthesis and crop productivity by accelerating recovery from photoprotection. Science (New York, N.Y.).

[CR105] Kunert R, Reinhart D (2016). Advances in recombinant antibody manufacturing. Appl Microbiol Biot.

[CR301] Kumar S, Worden A, Novak S, Lee R, Petolino JF (2016). A trait stacking system via intra-genomic homologous recombination. Planta.

[CR106] Langridge P (2014). Reinventing the green revolution by harnessing crop mutant resources. Plant Physiol.

[CR107] Lein W, Usadel B, Stitt M, Reindl A, Ehrhardt T, Sonnewald U, Börnke F (2008). Large-scale phenotyping of transgenic tobacco plants (*Nicotiana tabacum*) to identify essential leaf functions. Plant Biotechnol J.

[CR108] Lessard PA, Kulaveerasingam H, York GM, Strong A, Sinskey AJ (2002). Manipulating gene expression for the metabolic engineering of plants. Metab Eng.

[CR109] Li J-F, Zhang D, Sheen J (2014). Cas9-based genome editing in Arabidopsis and tobacco. Methods Enzymol.

[CR110] Li J, Meng X, Zong Y, Chen K, Zhang H, Liu J (2016). Gene replacements and insertions in rice by intron targeting using CRISPR-Cas9. Nat Plants.

[CR111] Li A, Jia S, Yobi A, Ge Z, Sato SJ, Zhang C (2018). Editing of an alpha-kafirin gene family increases, digestibility and protein quality in sorghum. Plant Physiol.

[CR112] Limkul J, Iizuka S, Sato Y, Misaki R, Ohashi T, Ohashi T, Fujiyama K (2016). The production of human glucocerebrosidase in glyco-engineered *Nicotiana benthamiana* plants. Plant Biotechnol J.

[CR113] Lin S, Zhang X, Song S, Hayat K, Eric K, Majeed H (2016). Tobacco alkaloids reduction by casings added/enzymatic hydrolysis treatments assessed through PLSR analysis. Regul Toxicol Pharmacol RTP.

[CR114] Liu W, Stewart CN (2016). Plant synthetic promoters and transcription factors. Curr Opin Biotechnol.

[CR115] Liu Y, Beyer A, Aebersold R (2016). On the dependency of cellular protein levels on mRNA abundance. Cell.

[CR116] Long SP, Marshall-Colon A, Zhu X-G (2015). Meeting the global food demand of the future by engineering crop photosynthesis and yield potential. Cell.

[CR117] Lowder LG, Paul JW, Qi Y (2017). Multiplexed transcriptional activation or repression in plants using CRISPR-dCas9-based systems. Methods Mol Biol.

[CR118] Lücker J, Schwab W, van Hautum B, Blaas J, van der Plas LHW, Bouwmeester HJ, Verhoeven HA (2004). Increased and altered fragrance of tobacco plants after metabolic engineering using three monoterpene synthases from lemon. Plant Physiol.

[CR119] Ludman M, Burgyán J, Fátyol K (2017). Crispr/Cas9 mediated inactivation of Argonaute 2 reveals its differential involvement in antiviral responses. Sci Rep.

[CR120] Lyzenga WJ, Harrington M, Bekkaoui D, Wigness M, Hegedus DD, Rozwadowski KL (2019). CRISPR/Cas9 editing of three CRUCIFERIN C homoeologues alters the seed protein profile in *Camelina sativa*. BMC Plant Biol.

[CR121] Ma JK, Drossard J, Lewis D, Altmann F, Boyle J, Christou P (2015). Regulatory approval and a first-in-human phase I clinical trial of a monoclonal antibody produced in transgenic tobacco plants. Plant Biotechnol J.

[CR122] Mahalik S, Sharma AK, Mukherjee KJ (2014). Genome engineering for improved recombinant protein expression in *Escherichia coli*. Microb Cell Fact.

[CR123] Mandal MK, Fischer R, Schillberg S, Schiermeyer A (2014). Inhibition of protease activity by antisense RNA improves recombinant protein production in *Nicotiana tabacum* cv. Bright Yellow 2 (BY-2) suspension cells. Biotechnol J.

[CR124] Mandal MK, Ahvari H, Schillberg S, Schiermeyer A (2016). Tackling unwanted proteolysis in plant production hosts used for molecular farming. Front Plant Sci.

[CR125] Margolin E, Oh YJ, Verbeek M, Naude J, Ponndorf D, Meshcheriakova YA (2020). Co-expression of human calreticulin significantly improves the production of HIV gp140 and other viral glycoproteins in plants. Plant Biotechnol J.

[CR126] Marin Viegas VS, Ocampo CG, Petruccelli S (2017). Vacuolar deposition of recombinant proteins in plant vegetative organs as a strategy to increase yields. Bioengineered.

[CR127] Marty F (1999). Plant vacuoles. Plant Cell.

[CR128] Matros A, Amme S, Kettig B, Buck-Sorlin GH, Sonnewald U, Mock H-P (2006). Growth at elevated CO2 concentrations leads to modified profiles of secondary metabolites in tobacco cv. SamsunNN and to increased resistance against infection with potato virus Y. Plant Cell Environ.

[CR129] Matsuo K, Atsumi G (2019). CRISPR/Cas9-mediated knockout of the RDR6 gene in *Nicotiana benthamiana* for efficient transient expression of recombinant proteins. Planta.

[CR130] Matsuo K, Matsumura T (2017). Repression of the DCL2 and DCL4 genes in *Nicotiana benthamiana* plants for the transient expression of recombinant proteins. J Biosci Bioeng.

[CR131] McNulty MJ, Gleba Y, Tusé D, Hahn-Löbmann S, Giritch A, Nandi S, McDonald KA (2020). Techno-economic analysis of a plant-based platform for manufacturing antimicrobial proteins for food safety. Biotechnol Prog.

[CR132] Mercx S, Smargiasso N, Chaumont F, de Pauw E, Boutry M, Navarre C (2017). Inactivation of the beta(1,2)-xylosyltransferase and the alpha(1,3)-fucosyltransferase genes in *Nicotiana tabacum* BY-2 cells by a multiplex CRISPR/Cas9 strategy results in glycoproteins without plant-specific glycans. Front Plant Sci.

[CR133] Miki D, Zhang W, Zeng W, Feng Z, Zhu J-K (2018). CRISPR/Cas9-mediated gene targeting in Arabidopsis using sequential transformation. Nat Commun.

[CR134] Mirondo R, Barringer S (2016). Deodorization of garlic breath by foods, and the role of polyphenol oxidase and phenolic compounds. J Food Sci.

[CR135] Montero-Morales L, Steinkellner H (2018). Advanced plant-based glycan engineering. Front Bioeng Biotechnol.

[CR136] Müller T, Thissen R, Braun S, Dott W, Fischer G (2004). (M)VOC and composting facilities. Part 2: (M)VOC dispersal in the environment. Environ Sci Pollut Res Int.

[CR137] Nadakuduti SS, Buell CR, Voytas DF, Starker CG, Douches DS (2018). Genome editing for crop improvement—applications in clonally propagated polyploids with a focus on potato (*Solanum tuberosum* L.). Front Plant Sci.

[CR138] Nagels B, van Damme EJM, Pabst M, Callewaert N, Weterings K (2011). Production of complex multiantennary *N*-glycans in *Nicotiana benthamiana* plants. Plant Physiol.

[CR139] Nandi S, Kwong AT, Holtz BR, Erwin RL, Marcel S, McDonald KA (2016). Techno-economic analysis of a transient plant-based platform for monoclonal antibody production. mAbs.

[CR140] Nedialkova DD, Leidel SA (2015). Optimization of codon translation rates via tRNA modifications maintains proteome integrity. Cell.

[CR141] Ocampo CG, Lareu JF, Marin Viegas VS, Mangano S, Loos A, Steinkellner H, Petruccelli S (2016). Vacuolar targeting of recombinant antibodies in *Nicotiana benthamiana*. Plant Biotechnol J.

[CR142] Oey M, Lohse M, Kreikemeyer B, Bock R (2009). Exhaustion of the chloroplast protein synthesis capacity by massive expression of a highly stable protein antibiotic. Plant J.

[CR143] Oono Y, Wakasa Y, Hirose S, Yang L, Sakuta C, Takaiwa F (2010). Analysis of ER stress in developing rice endosperm accumulating beta-amyloid peptide. Plant Biotechnol J.

[CR144] Opdensteinen P, Clodt JI, Müschen CR, Filiz V, Buyel JF (2018). A combined ultrafiltration/diafiltration step facilitates the purification of cyanovirin-N from transgenic tobacco extracts. J Biotechnol.

[CR145] Panganiban RA, Park H-R, Sun M, Shumyatcher M, Himes BE, Lu Q (2019). Genome-wide CRISPR screen identifies suppressors of endoplasmic reticulum stress-induced apoptosis. Proc Natl Acad Sci U S A.

[CR146] Park Y, Runkle ES (2018). Spectral effects of light-emitting diodes on plant growth, visual color quality, and photosynthetic photon efficacy: white versus blue plus red radiation. PLoS ONE.

[CR147] Parsons J, Altmann F, Arrenberg CK, Koprivova A, Beike AK, Stemmer C (2012). Moss-based production of asialo-erythropoietin devoid of Lewis A and other plant-typical carbohydrate determinants. Plant Biotechnol J.

[CR148] Parsons J, Altmann F, Graf M, Stadlmann J, Reski R, Decker EL (2013). A gene responsible for prolyl-hydroxylation of moss-produced recombinant human erythropoietin. Sci Rep.

[CR149] Pastor-Cantizano N, Ko DK, Angelos E, Pu Y, Brandizzi F (2020). Functional diversification of ER stress responses in Arabidopsis. Trends Biochem Sci.

[CR150] Pierik R, Voesenek LACJ, de Kroon H, Visser EJW (2004). Density-induced plant size reduction and size inequalities in ethylene-sensing and ethylene-insensitive tobacco. Plant Biol (Stuttgart, Germany).

[CR151] Pillay P, Kibido T, Du Plessis M, van der Vyver C, Beyene G, Vorster BJ (2012). Use of transgenic oryzacystatin-I-expressing plants enhances recombinant protein production. Appl Biochem Biotechnol.

[CR152] Piron R, Santens F, de Paepe A, Depicker A, Callewaert N (2015). Using GlycoDelete to produce proteins lacking plant-specific *N*-glycan modification in seeds. Nat Biotechnol.

[CR153] Poorter H, Niklas KJ, Reich PB, Oleksyn J, Poot P, Mommer L (2012). Biomass allocation to leaves, stems and roots: meta-analyses of interspecific variation and environmental control. New Phytol.

[CR196] Puchol Tarazona AA, Lobner E, Taubenschmid Y, Paireder M, Torres Acosta JA, Göritzer K (2020). Steric accessibility of the cleavage sites dictates the proteolytic vulnerability of the anti-HIV-1 antibodies 2F5, 2G12, and PG9 in plants. Biotechnol J.

[CR154] Rajeevkumar S, Anunanthini P, Sathishkumar R (2015). Epigenetic silencing in transgenic plants. Front Plant Sci.

[CR155] Raschmanová H, Zamora I, Borčinová M, Meier P, Weninger A, Mächler D (2019). Single-cell approach to monitor the unfolded protein response during biotechnological processes with *Pichia pastoris*. Front Microbiol.

[CR156] Revuelta-Aramburu M, Verdú-Vázquez A, Gil-López T, Morales-Polo C (2020). Environmental analysis of the use of plant fiber blocks in building construction. Sci Total Environ.

[CR157] Robert S, Goulet M-C, D'Aoust M-A, Sainsbury F, Michaud D (2015). Leaf proteome rebalancing in *Nicotiana benthamiana* for upstream enrichment of a transiently expressed recombinant protein. Plant Biotechnol J.

[CR158] Robson PR, McCormac AC, Irvine AS, Smith H (1996). Genetic engineering of harvest index in tobacco through overexpression of a phytochrome gene. Nat Biotechnol.

[CR159] Roslan HA, Salter MG, Wood CD, White MR, Croft KP, Robson F (2001). Characterization of the ethanol-inducible alc gene-expression system in *Arabidopsis thaliana*. Plant J.

[CR160] Sack M, Rademacher T, Spiegel H, Boes A, Hellwig S, Drossard J (2015). From gene to harvest. Insights into upstream process development for the GMP production of a monoclonal antibody in transgenic tobacco plants. Plant Biotechnol J.

[CR161] Sahoo S, Das SS, Rakshit R (2019). Codon usage pattern and predicted gene expression in *Arabidopsis thaliana*. Gene X.

[CR162] Sainsbury F, Varennes-Jutras P, Goulet M-C, D'Aoust M-A, Michaud D (2013). Tomato cystatin SlCYS8 as a stabilizing fusion partner for human serpin expression in plants. Plant Biotechnol J.

[CR163] Salter MG, Paine JA, Riddell KV, Jepson I, Greenland AJ, Caddick MX, Tomsett AB (1998). Characterisation of the ethanol-inducible alc gene expression system for transgenic plants. Plant J.

[CR164] Sánchez-León S, Gil-Humanes J, Ozuna CV, Giménez MJ, Sousa C, Voytas DF, Barro F (2018). Low-gluten, nontransgenic wheat engineered with CRISPR/Cas9. Plant Biotechnol J.

[CR165] Schaarschmidt S, Qu N, Strack D, Sonnewald U, Hause B (2004). Local induction of the alc gene switch in transgenic tobacco plants by acetaldehyde. Plant Cell Physiol.

[CR166] Schachtsiek J, Stehle F (2019). Nicotine-free, nontransgenic tobacco (*Nicotiana tabacum* L.) edited by CRISPR-Cas9. Plant Biotechnol J.

[CR167] Schiavon M, Martini LM, Corrà C, Scapinello M, Coller G, Tosi P, Ragazzi M (2017). Characterisation of volatile organic compounds (VOCs) released by the composting of different waste matrices. Environ Pollut (Barking, Essex: 1987).

[CR168] Schiermeyer A (2020). Optimizing product quality in molecular farming. Curr Opin Biotechnol.

[CR169] Schiermeyer A, Schneider K, Kirchhoff J, Schmelter T, Koch N, Jiang K (2019). Targeted insertion of large DNA sequences by homology-directed repair or non-homologous end joining in engineered tobacco BY-2 cells using designed zinc finger nucleases. Plant Direct.

[CR170] Schmidt FJ, Zimmermann MM, Wiedmann DR, Lichtenauer S, Grundmann L, Muth J (2019). The major floral promoter NtFT5 in Tobacco (*Nicotiana tabacum*) is a promising target for crop improvement. Front Plant Sci.

[CR171] Schmidt JA, McGrath JM, Hanson MR, Long SP, Ahner BA (2019). Field-grown tobacco plants maintain robust growth while accumulating large quantities of a bacterial cellulase in chloroplasts. Nat Plants.

[CR172] Schneider K, Schiermeyer A, Dolls A, Koch N, Herwartz D, Kirchhoff J (2016). Targeted gene exchange in plant cells mediated by a zinc finger nuclease double cut. Plant Biotechnol J.

[CR173] Schoberer J, Strasser R (2018). Plant glyco-biotechnology. Semin Cell Dev Biol.

[CR174] Schuck S, Prinz WA, Thorn KS, Voss C, Walter P (2009). Membrane expansion alleviates endoplasmic reticulum stress independently of the unfolded protein response. J Cell Biol.

[CR175] Shaaltiel Y, Bartfeld D, Hashmueli S, Baum G, Brill-Almon E, Galili G (2007). Production of glucocerebrosidase with terminal mannose glycans for enzyme replacement therapy of Gaucher's disease using a plant cell system. Plant Biotechnol J.

[CR176] Shah P, Gilchrist MA (2011). Explaining complex codon usage patterns with selection for translational efficiency, mutation bias, and genetic drift. Proc Natl Acad Sci U S A.

[CR177] Shao M, Michno J-M, Hotton SK, Blechl A, Thomson J (2015). A bacterial gene codA encoding cytosine deaminase is an effective conditional negative selectable marker in *Glycine max*. Plant Cell Rep.

[CR178] Sharma AK, Shukla E, Janoti DS, Mukherjee KJ, Shiloach J (2020). A novel knock out strategy to enhance recombinant protein expression in *Escherichia coli*. Microb Cell Fact.

[CR179] Sheen SJ (1983). Biomass and chemical composition of tobacco plants under high density growth. Beiträge zur Tabakforschung/Contributions to Tobacco Research.

[CR180] Shigemitsu T, Ozaki S, Saito Y, Kuroda M, Morita S, Satoh S, Masumura T (2012). Production of human growth hormone in transgenic rice seeds: co-introduction of RNA interference cassette for suppressing the gene expression of endogenous storage proteins. Plant Cell Rep.

[CR181] Shin Y-J, Castilho A, Dicker M, Sádio F, Vavra U, Grünwald-Gruber C (2017). Reduced paucimannosidic *N*-glycan formation by suppression of a specific β-hexosaminidase from *Nicotiana benthamiana*. Plant Biotechnol J.

[CR182] Shoji Y, Farrance CE, Bautista J, Bi H, Musiychuk K, Horsey A (2012). A plant-based system for rapid production of influenza vaccine antigens. Influenza Other Resp.

[CR183] Song EG, Ryu KH (2017). A pepper mottle virus-based vector enables systemic expression of endoglucanase D in non-transgenic plants. Arch Virol.

[CR184] Sourrouille C, Marquet-Blouin E, D'Aoust M-A, Kiefer-Meyer M-C, Seveno M, Pagny-Salehabadi S (2008). Down-regulated expression of plant-specific glycoepitopes in alfalfa. Plant Biotechnol J.

[CR185] South PF, Cavanagh AP, Liu HW, Ort DR (2019). Synthetic glycolate metabolism pathways stimulate crop growth and productivity in the field. Science (New York, N.Y.).

[CR186] Strasser R (2016). Plant protein glycosylation. Glycobiology.

[CR187] Strasser R (2018). Protein quality control in the endoplasmic reticulum of plants. Annu Rev Plant Biol.

[CR188] Strasser R, Stadlmann J, Schahs M, Stiegler G, Quendler H, Mach L (2008). Generation of glyco-engineered *Nicotiana benthamiana* for the production of monoclonal antibodies with a homogeneous human-like *N*-glycan structure. Plant Biotechnol J.

[CR189] Strasser R, Castilho A, Stadlmann J, Kunert R, Quendler H, Gattinger P (2009). Improved virus neutralization by plant-produced anti-HIV antibodies with a homogeneous beta1,4-galactosylated *N*-glycan profile. J Biol Chem.

[CR190] Suo G, Chen B, Zhang J, Duan Z, He Z, Yao W (2006). Effects of codon modification on human BMP2 gene expression in tobacco plants. Plant Cell Rep.

[CR191] Svitashev S, Young JK, Schwartz C, Gao H, Falco SC, Cigan AM (2015). Targeted mutagenesis, precise gene editing, and site-specific gene insertion in maize using Cas9 and guide RNA. Plant Physiol.

[CR192] Sweetman JP, Chu C, Qu N, Greenland AJ, Sonnewald U, Jepson I (2002). Ethanol vapor is an efficient inducer of the alc gene expression system in model and crop plant species. Plant Physiol.

[CR193] Tada Y, Utsumi S, Takaiwa F (2003). Foreign gene products can be enhanced by introduction into low storage protein mutants. Plant Biotechnol J.

[CR194] Takaiwa F (2013). Increasing the production yield of recombinant protein in transgenic seeds by expanding the deposition space within the intracellular compartment. Bioengineered.

[CR195] Takaiwa F, Wakasa Y, Hayashi S, Kawakatsu T (2017). An overview on the strategies to exploit rice endosperm as production platform for biopharmaceuticals. Plant Sci Int J Exp Plant Biol.

[CR197] Tegel H, Tourle S, Ottosson J, Persson A (2010). Increased levels of recombinant human proteins with the *Escherichia coli* strain Rosetta (DE3). Protein Expr Purif.

[CR198] Tewolde FT, Shiina K, Maruo T, Takagaki M, Kozai T, Yamori W (2018). Supplemental LED inter-lighting compensates for a shortage of light for plant growth and yield under the lack of sunshine. PLoS ONE.

[CR199] Thomas DR, Walmsley AM (2015). The effect of the unfolded protein response on the production of recombinant proteins in plants. Plant Cell Rep.

[CR200] Torrent M, Chalancon G, de Groot NS, Wuster A, Madan Babu M (2018). Cells alter their tRNA abundance to selectively regulate protein synthesis during stress conditions. Sci Signal.

[CR201] Tuller T, Carmi A, Vestsigian K, Navon S, Dorfan Y, Zaborske J (2010). An evolutionarily conserved mechanism for controlling the efficiency of protein translation. Cell.

[CR202] Tusé D, Nandi S, McDonald KA, Buyel JF (2020). The emergency response capacity of plant-based biopharmaceutical manufacturing-what it is and what it could be. Front Plant Sci.

[CR203] Twyman RM, Stoger E, Schillberg S, Christou P, Fischer R (2003). Molecular farming in plants. Host systems and expression technology. Trends Biotechnol.

[CR204] ul Hassan MN, Zainal Z, Ismail I (2015). Green leaf volatiles: biosynthesis, biological functions and their applications in biotechnology. Plant Biotechnol J.

[CR205] Urquidi Camacho RA, Lokdarshi A, von Arnim AG (2020). Translational gene regulation in plants: a green new deal. Wiley Interdiscip Rev RNA.

[CR206] van der Hoorn RAL (2008). Plant proteases: from phenotypes to molecular mechanisms. Annu Rev Plant Biol.

[CR207] Vicuna Requesens D, Gonzalez Romero ME, Devaiah SP, Chang Y-K, Flory A, Streatfield S (2019). The maize α-zein promoter can be utilized as a strong inducer of cellulase enzyme expression in maize kernels. Transgenic Res.

[CR208] Wakasa Y, Yasuda H, Oono Y, Kawakatsu T, Hirose S, Takahashi H (2011). Expression of ER quality control-related genes in response to changes in BiP1 levels in developing rice endosperm. Plant J Cell Mol Biol.

[CR302] Wakasa Y, Hayashi S, Ozawa K, Takaiwa F (2012). Multiple roles of the ER stress sensor IRE1 demonstrated by gene targeting in rice. Sci Rep.

[CR209] Walwyn DR, Huddy SM, Rybicki EP (2015). Techno-Economic analysis of horseradish peroxidase production using a transient expression system in *Nicotiana benthamiana*. Appl Biochem Biotechnol.

[CR210] Wang D, Ma J, Sun D, Li H, Jiang C, Li X (2015). Expression of bioactive anti-CD20 antibody fragments and induction of ER stress response in Arabidopsis seeds. Appl Microbiol Biotechnol.

[CR211] Wang X, Ye L, Lyu M, Ursache R, Löytynoja A, Mähönen AP (2020). An inducible genome editing system for plants. Nat Plants.

[CR212] Warren JM, Salinas-Giegé T, Hummel G, Coots NL, Svendsen JM, Brown KC (2020). Combining tRNA sequencing methods to characterize plant tRNA expression and post-transcriptional modification. RNA Biol.

[CR213] Watanabe K, Breier U, Hensel G, Kumlehn J, Schubert I, Reiss B (2016). Stable gene replacement in barley by targeted double-strand break induction. J Exp Bot.

[CR214] Webster GR, Teh AY-H, Ma JK-C (2017). Synthetic gene design—the rationale for codon optimization and implications for molecular pharming in plants. Biotechnol Bioeng.

[CR215] Weinthal DM, Taylor RA, Tzfira T (2013). Nonhomologous end joining-mediated gene replacement in plant cells. Plant Physiol.

[CR216] Weise A, Altmann F, Rodriguez-Franco M, Sjoberg ER, Bäumer W, Launhardt H (2007). High-level expression of secreted complex glycosylated recombinant human erythropoietin in the Physcomitrella Delta-fuc-t Delta-xyl-t mutant. Plant Biotechnol J.

[CR217] Wolter F, Puchta H (2018). The CRISPR/Cas revolution reaches the RNA world: Cas13, a new Swiss Army knife for plant biologists. Plant J.

[CR218] Xiao Y, He X, Ojeda-Lassalle Y, Poovaiah C, Coleman HD (2018). Expression of a hyperthermophilic endoglucanase in hybrid poplar modifies the plant cell wall and enhances digestibility. Biotechnol Biofuels.

[CR219] Xiong TC, Sanchez F, Briat J-F, Gaymard F, Dubos C (2016). Spatio-temporal imaging of promoter activity in intact plant tissues. Methods Mol Biol (Clifton, N.J.).

[CR220] Yamamoto T, Hoshikawa K, Ezura K, Okazawa R, Fujita S, Takaoka M (2018). Improvement of the transient expression system for production of recombinant proteins in plants. Sci Rep.

[CR221] Yang S-J, Carter SA, Cole AB, Cheng N-H, Nelson RS (2004). A natural variant of a host RNA-dependent RNA polymerase is associated with increased susceptibility to viruses by Nicotiana benthamiana. Proc Natl Acad Sci U S A.

[CR222] Yang L, Hirose S, Takahashi H, Kawakatsu T, Takaiwa F (2012). Recombinant protein yield in rice seed is enhanced by specific suppression of endogenous seed proteins at the same deposit site. Plant Biotechnol J.

[CR223] Yang WC, Minkler DF, Kshirsagar R, Ryll T, Huang YM (2016). Concentrated fed-batch cell culture increases manufacturing capacity without additional volumetric capacity. J Biotechnol.

[CR224] Yoon S, Devaiah SP, Choi S, Bray J, Love R, Lane J (2016). Over-expression of the cucumber expansin gene (Cs-EXPA1) in transgenic maize seed for cellulose deconstruction. Transgenic Res.

[CR225] Yuki Y, Mejima M, Kurokawa S, Hiroiwa T, Kong IG, Kuroda M (2012). RNAi suppression of rice endogenous storage proteins enhances the production of rice-based *Botulinum* neutrotoxin type A vaccine. Vaccine.

[CR226] Zhao D, Hamilton JP, Hardigan M, Yin D, He T, Vaillancourt B (2017). Analysis of ribosome-associated mRNAs in rice reveals the importance of transcript size and GC content in translation. G3 (Bethesda, MD).

[CR227] Zhong S-H, Liu J-Z, Jin H, Lin L, Li Q, Chen Y (2013). Warm temperatures induce transgenerational epigenetic release of RNA silencing by inhibiting siRNA biogenesis in Arabidopsis. Proc Natl Acad Sci U S A.

[CR228] Zhou X-R, Bhandari S, Johnson BS, Kotapati HK, Allen DK, Vanhercke T, Bates PD (2020). Reorganization of acyl flux through the lipid metabolic network in oil-accumulating tobacco leaves. Plant Physiol.

[CR229] Zhu H, Bhatt B, Sivaprakasam S, Cai Y, Liu S, Kodeboyina SK (2019). Ufbp1 promotes plasma cell development and ER expansion by modulating distinct branches of UPR. Nature Commun.

[CR230] Zischewski J, Sack M, Fischer R (2015). Overcoming low yields of plant-made antibodies by a protein engineering approach. Biotechnol J.

